# Pharmacological effects and therapeutic potential of natural compounds in neuropsychiatric disorders: An update

**DOI:** 10.3389/fphar.2022.926607

**Published:** 2022-09-15

**Authors:** Parina Asgharian, Cristina Quispe, Jesús Herrera-Bravo, Mahsa Sabernavaei, Kamran Hosseini, Haleh Forouhandeh, Tahereh Ebrahimi, Paria Sharafi-Badr, Vahideh Tarhriz, Saiedeh Razi Soofiyani, Paweł Helon, Jovana Rajkovic, Sevgi Durna Daştan, Anca Oana Docea, Javad Sharifi-Rad, Daniela Calina, Wojciech Koch, William C. Cho

**Affiliations:** ^1^ Drug Applied Research Center, Tabriz University of Medical Sciences, Tabriz, Iran; ^2^ Facultad de Ciencias de la Salud, Universidad Arturo Prat, Iquique, Chile; ^3^ Departamento de Ciencias Básicas, Facultad de Ciencias, Universidad Santo Tomas, Santo Tomas, Chile; ^4^ Center of Molecular Biology and Pharmacogenetics, Scientific and Technological Bioresource Nucleus, Universidad de La Frontera, Temuco, Chile; ^5^ Department of Pharmacognosy and Pharmaceutical Biotechnology, School of Pharmacy, Iran University of Medical Sciences, Tehran, Iran; ^6^ Student Research Committee, Shiraz University of Medical Sciences, Shiraz, Iran; ^7^ Department of Molecular Medicine, Faculty of Advanced Medical Sciences and Technologies, Shiraz University of Medical Sciences, Shiraz, Iran; ^8^ Infectious and Tropical Diseases Research Center, Biomedicine Institute, Tabriz University of Medical Sciences, Tabriz, Iran; ^9^ Department of Pharmacognosy, School of Pharmacy, Tehran University of Medical Sciences, Tehran, Iran; ^10^ Clinical Research Development Unit of Sina Educational, Research and Treatment Center, Tabriz University of Medical Sciences, Tabriz, Iran; ^11^ Branch in Sandomierz, Jan Kochanowski University of Kielce, Sandomierz, Poland; ^12^ Medical Faculty, Institute of Pharmacology, Clinical Pharmacology and Toxicology, University of Belgrade, Belgrade, Serbia; ^13^ Department of Biology, Faculty of Science, Sivas Cumhuriyet University, Sivas, Turkey; ^14^ Beekeeping Development Application and Research Center, Sivas Cumhuriyet University, Sivas, Turkey; ^15^ Department of Toxicology, University of Medicine and Pharmacy of Craiova, Craiova, Romania; ^16^ Facultad de Medicina, Universidad del Azuay, Cuenca, Ecuador; ^17^ Department of Clinical Pharmacy, University of Medicine and Pharmacy of Craiova, Craiova, Romania; ^18^ Department of Food and Nutrition, Medical University of Lublin, Lublin, Poland; ^19^ Department of Clinical Oncology, Queen Elizabeth Hospital, Kowloon, Hong Kong SAR, China

**Keywords:** neuropsychiatric disorders, natural compounds, pharmacological mechanisms, bioactive compounds, preclinical pharmacology

## Abstract

Neuropsychiatric diseases are a group of disorders that cause significant morbidity and disability. The symptoms of psychiatric disorders include anxiety, depression, eating disorders, autism spectrum disorders (ASD), attention-deficit/hyperactivity disorder, and conduct disorder. Various medicinal plants are frequently used as therapeutics in traditional medicine in different parts of the world. Nowadays, using medicinal plants as an alternative medication has been considered due to their biological safety. Despite the wide range of medications, many patients are unable to tolerate the side effects and eventually lose their response. By considering the therapeutic advantages of medicinal plants in the case of side effects, patients may prefer to use them instead of chemical drugs. Today, the use of medicinal plants in traditional medicine is diverse and increasing, and these plants are a precious heritage for humanity. Investigation about traditional medicine continues, and several studies have indicated the basic pharmacology and clinical efficacy of herbal medicine. In this article, we discuss five of the most important and common psychiatric illnesses investigated in various studies along with conventional therapies and their pharmacological therapies. For this comprehensive review, data were obtained from electronic databases such as MedLine/PubMed, Science Direct, Web of Science, EMBASE, DynaMed Plus, ScienceDirect, and TRIP database. Preclinical pharmacology studies have confirmed that some bioactive compounds may have beneficial therapeutic effects in some common psychiatric disorders. The mechanisms of action of the analyzed biocompounds are presented in detail. The bioactive compounds analyzed in this review are promising phytochemicals for adjuvant and complementary drug candidates in the pharmacotherapy of neuropsychiatric diseases. Although comparative studies have been carefully reviewed in the preclinical pharmacology field, no clinical studies have been found to confirm the efficacy of herbal medicines compared to FDA-approved medicines for the treatment of mental disorders. Therefore, future clinical studies are needed to accelerate the potential use of natural compounds in the management of these diseases.

## 1 Introduction

Neuropsychiatric disorders are a group of disorders that cause great morbidity and disability. Globally, the psychiatric disorder’s prevalence is estimated at 6.7%. The symptoms of psychiatric disorders include anxiety, depression, eating disorders, autism spectrum, attention-deficit/hyperactivity, and conduct disorder. Different studies have been probed to clarify the basic molecular mechanism involved in such a disease’s occurrence. Recently, it has been shown that early-life experiences can affect adulthood behaviour. Nurturance, genetics, and environment are important factors that influence behaviour in adulthood. Like other multifactorial disorders, non-genetic factors are important factors in the aetiology of this condition (Martens and van loo, 2007; Cannon and Greenamyre, 2011).

Neuropsychiatric disorders are dealing with mental and cerebral disorders often associated with brain dysfunction (Yudofsky and Hales, 2002; Nussbaum et al., 2017). Many researchers use beneficial therapies with the least side effects to treat these patients. Therefore, choosing the right type of treatment depends on the variety of diseases that the person is suffering from (Reddy et al., 2020). Patients with any brain injury are more sensitive to the side effects of chemical drugs than patients without injury. Therefore, the physician should be careful in choosing the appropriate type of medication, dose, and duration of treatment (Silver et al., 1990; Silver et al., 1991; Silver et al., 1994). Numerous studies on animal models have shown that some chemical drugs, such as haloperidol, benzodiazepines, and clonidines, may interfere with the recovery of neuronal damage and eventually disrupt the normal physiological processes in the brain (Kuhn et al., 2019). Current medications for neuropsychiatric diseases mainly target disease symptoms. Therefore, there is a critical necessity to develop therapeutics which can delay, stop or reverse the progression of the condition (Paul and Snyder, 2019).

Clinical studies use antioxidants to interfere in disease progression, but the results are not satisfactory. Most of the antioxidants non-specifically target neuroprotective pathways. Consequently, new studies are needed to discover new potential agents that restore redox balance along with reducing neuronal damage (Underwood et al., 2010). Nowadays, using medicinal plants as an alternative medication has been considered due to their biological safety (Quetglas-Llabrés et al., 2022). In this article, we discuss the most important and common psychiatric illnesses mentioned in various studies along with conventional therapies and their pharmacological therapies.

## 2 Search methodology

For this comprehensive review, data were obtained from electronic databases such as MedLine/PubMed, Science Direct, Web of Science, EMBASE, DynaMed Plus, ScienceDirect, and TRIP database. The following MeSH terms were used for the search: “Plants, Medicinal”, “Antidepressive Agents/isolation and purification,” “Antidepressive Agents/pharmacology,” “Action Potentials/drug effects,” “Animals,” “Disease Models,” “Animal, Plant Bark/chemistry,” “Plant Extracts/chemistry,” “Serotonin/metabolism,” “Synapsis agonists,” “Brain/drug effects,” “Brain/metabolism,” “Seizures/prevention and control,” “Attention Deficit Disorder with Hyperactivity/drug therapy,” “Phytotherapy/methods,” “Phytotherapy/adverse effects,” “Evidence-Based Medicine,” “Treatment Outcome,” “Autism/natural products/treatment,” “schizophrenia/natural products/treatment.” Preclinical pharmacological studies were included to explain the effects and potential mechanisms of natural bioactive compounds in some common neuropsychiatric disorders. Only papers written in English that included the potential mechanisms of natural compounds in psychiatric disorders were selected. The plants’ taxonomy has been validated according to PlantList (Heinrich et al., 2020; Plantlist, 2021). Duplicate papers, communications, and studies that included homeopathic preparations or other brain conditions such as tumors were excluded.

## 3 Treatment of neuropsychiatric disorders in conventional meaning, using approved drugs and bioactive compounds: Underlying potential mechanisms

### 3.1 Major depressive disorder

Major depressive disorder (MDD) is identified by two characteristics: depressive state in several conditions and apathy with somatic and cognitive disturbances (World Health Organization, 1992; Otte et al., 2016; Vlad et al., 2020). The most common time of onset is between the ages of 20 and 30, and women are twice as likely as men to be affected (American Psychiatric Association, 1980; Wulff et al., 2015). Its lifetime prevalence is 16.6% per person (Weissman and Olfson, 1995; Kessler et al., 2005). The physiopathology of the disease is not yet clear, but it is associated with abnormalities in the brain’s monoamine receptors or neurotransmitters, metinflammation conditions and as well as the serotonergic, noradrenergic, and neuropeptide systems are abnormal (Manji et al., 2001; Charney and Manji, 2004). Numerous studies have shown that the hypothalamic-pituitary-adrenal (HPA) axis is involved in this process and contributes to neuronal atrophy (Nestler et al., 2002; Mann and Currier, 2006).

#### 3.1.1 Treatment of major depressive disorder using approved drugs

Conventional disease treatments include lifestyle changes such as exercise and smoking cessation (Goldberg et al., 2005; Taylor et al., 2014), somatic treatments such as electroconvulsive therapy (effective in resistant depression) (Paul et al., 1981; Prudic et al., 1996), focused psychotherapies (such as relaxation and mindfulness, behavioural therapy, and interpersonal therapy) (DeRubeis et al., 2005), and pharmacotherapy.

Pharmacotherapeutic therapies include selective serotonin reuptake inhibitors (SSRIs) such as citalopram, escitalopram, paroxetine, etc. (Papakostas, 2010); serotonin-norepinephrine reuptake inhibitors (SNRIs) such as venlafaxine (Stahl et al., 2005); tricyclic antidepressants such as ampitripitillin, clomipramine, doxepine, etc. (Moore and O’Keeffe, 1999); and monoamine oxidase inhibitors (MAOIs) such as phenelzine, vortioxetine and others (Table 1 (Quitkin et al., 1984; Quitkin et al., 1988).

**TABLE 1:** Approved drugs and their biological function in the treatment of important neuropsychiatric disorders.

Disease	Main group of drugs	Biological functional	References
MDD	Citalopram (Celexa)	Serotonin reuptake inhibitors (SSRIs)	(Fava et al., 2004; Papakostas, 2010; Ravindran and Stein, 2010)
	Escitalopram (Lexapro)		
	Paroxetine (Paxil, Paxil CR)		
	Sertraline (Zoloft)		
	Fluvoxamine (Luvox)		
	Fluoxetine (Prozac)		
	Venlafaxine (Effexor, Effexor XR)	Serotonin-norepinephrine reuptake inhibitors (SNRIs)	Stahl et al. (2005)
	Desvenlafaxine (Pristiq)		
	Duloxetine (Cymbalta)		
	Amitriptyline (Elavil)	Blocking the activity of serotonin 5-HT2 receptors	(Snyder and Yamamura, 1977; Preskorn and Simpson, 1982; Lavoie et al., 1990; Atkinson et al., 1998; Moore and O’Keeffe, 1999; Menza et al., 2009)
	Clomipramine (Anafranil)		
	Doxepin (Adapin)		
	Imipramine (Tofranil)		
	Trimipramine (Surmontil) Desipramine (Norpramin) Nortriptyline (Pamelor) Protriptyline (Vivactil)		
	Amoxapine (Asendin)		
	Maprotiline (Ludiomil)		
	Phenelzine (Nardil) Tranylcypromine (Parnate) Isocarboxazid (Marplan)	Monoamine oxidase inhibitors (MAOIs)	(Quitkin et al., 1984; Quitkin et al., 1988; Lane and Baldwin, 1997; Association, 2006)
	Selegiline (Eldepryl)		
	Selegiline transdermal (Emsam)		
Schizophrenia	First-generation antipsychotics (Phenothiazines, Butyrophenones, Thioxanthenes, Dihydroindolones, Dibenzepines, Diphenylbutylpiperidines)	Dopamine antagonist (Blocking dopamine receptors)	Freedman, (2010)
	Second-generation antipsychotics (clozapine, olanzapine, quetiapine, risperidone, paliperidone, ziprasidone, and molindone	Serotonin-Dopamine Antagonists (D2, 5-HT1A, and 5-HT2A receptors)	(Gupta et al., 1994; Seeger et al., 1995; Möller, 2005; Schmid et al., 2014; Brenner and Stevens, 2017)
	Third-generation antipsychotics (aripiprazole, brexpiprazole and cariprazine)	D2 partial agonists	(Burris et al., 2002; Shapiro et al., 2003; De Deurwaerdère, 2016; Hope et al., 2018)
Autism	Risperidone	Serotonin-Dopamine Antagonists	(Leskovec et al., 2008; Rapin and Tuchman, 2008; Ji and Findling, 2015)
	Aripiprazole		
	Fluoxetine and fluvoxamine	Serotonin reuptake inhibitors (SSRIs)	Johnson and Myers, (2007)
	Methylphenidate	Norepinephrine—dopamine reuptake inhibitor (NDRI)	
Bipolar Disorder	mood stabilizers (Lithium, Divalproex, Carbamazepine)	↓ norepinephrine release and increasing serotonin synthesis	(Allen et al., 2006; Malhi et al., 2009; Miura et al., 2014)
	antipsychotic drugs (aripiprazole, Quetiapine, Risperidone, Olanzapine, Paliperidone)	Blocking dopamine D2 receptors	Jain, (2020)
ADHD	Methylphenidate	Norepinephrine—dopamine reuptake inhibitor (NDRI)	Storebø et al. (2015)
	Viloxazine	Norepinephrine reuptake inhibitor	Banaschewski et al. (2004)
	Atomoxetine	Norepinephrine reuptake inhibitor	
	Bupropion	Norepinephrine–dopamine reuptake inhibitor (NDRI) and antagonist of several nicotinic acetylcholine receptors	
	Guanfacine	Activating α_2A_ adrenoceptors	
	clonidine	Agonist of alpha-2A adrenergic receptor	
Epilepsy	Phenytoin	Sodium channel blocker	(Nevitt et al., 2018; Nevitt et al., 2019)
	Carbamazepine		Uk, (2012)
	Valproate		
	Lamotrigine		
	Levetiracetam		
	Phenobarbital	↑chloride ions into post-synaptic neuron s	
		↓excitability of the neurons	Newton and Garcia, (2012)

#### 3.1.2 Treatment of major depressive disorder and bioactive compounds

MDD is a significant prospect of global mental and economic burden. In most patients, the specific clinical features following symptoms such as sleep dysregulation, depressed mood, fatigue, suicidal thoughts, and loss of interest and appetite are observed (Yeni et al., 2022). The change in serotonin, norepinephrine and dopamine levels has been linked to clinical symptoms based on the monoamine hypothesis (Shyn and Hamilton, 2010; Willner et al., 2013).

Some plants are effective in modifying the mood by the effect on the monoamine neurotransmission, similar to *Hypericum perforatum*, as well as have an impact on GABA, opioid, and cannabinoid systems (Table 2) (Spinella, 2001; Heinrich et al., 2017).

**TABLE 2:** Summarizes the effects and potential effects for the most important phytochemicals as a promising therapy for treating major depressive disorders.

Compounds	Main group of compounds	Verified effective concentrations/model	Potential effects	References
Alkaloids	membrane-like alkaloids	Dose = 25 mg randomized double-blind placebo-controlled study	↑amygdala response to scary facial expressions	(Chiu et al., 2014) (Chiu et al., 2017) (Gericke and Van Wyk, 2001b) (Napoletano et al., 2001; Houslay et al., 2005)
			↑serotonin	
			↓cAMP	
	Curcumin	Dose = 5–10 mg/kg mice	↑NA	(Xu et al., 2005b; Darvesh et al., 2012)
			↑serotonin in the frontal cortex and hippocampal brain	Xu et al. (2007)
			↓MAO-A, ↓MAO-B	Wang et al. (2008)
Phenolic Phytochemicals		*in vivo*	↑hippocampal neurogenesis	Li et al. (2009)
			Modulation of the serotoninergic system	Lin et al. (2011)
			↓AC/cAMP, ↓cAMP	(Wang et al., 2008; Wang et al., 2010)
			↓glutamate	Kulkarni et al. (2008)
			↑neurotrophic factors	
			↑serotonin, ↑dopamine	
	Amentoflavone	Dose = 6.25–50 mg/kg mice	↓immobility inhibition flumazenil binding to GABA receptor	Ishola et al. (2012), Baureithel et al. (1997)
		*in vivo*		
	Chlorogenic acid	Dose = 200–400 mg/kg mice	↓MAOB, ↓ ROS	(Wu et al., 2016; Lim et al., 2018) (Park et al., 2010) (Chen et al., 2021)
		*in vivo*	↑ axon and dendrite growth	
			↑serotonin release through enhancing synapsin expression act through the opioidergic pathway	
			↑ neuroinflammation and oxidative stress	
	Ellagic acid	Dose = 25–100 mg/kg mice	↓immobility period in both FST and TST effect in monoaminergic neurotransmitter receptors	Girish et al. (2012)
		*in vivo*		
	Ferulic acid	Dose = 0.01–10 mg/kg mice	↓ serotonin reuptake anti-inflammatory	(Zeni et al., 2012) (Sasaki et al., 2019)
		*in vivo*	antioxidant	
			neuroprotective	
	Fisetin	Dose = 10–25 mg/kg mice	↓MAO	(Zheng et al., 2008; Zhen et al., 2012; Yao et al., 2020)
		*in vivo*	↓5-HT, ↓NA, ↓DA reuptake	
			↓oxido-nitrosative stress, ↓ROS, anti-inflammatory effect	
	Quercetin	Dose = 50–100 mg/kg mice	depression-like effect through the participation of α2 adrenergic receptors in its mechanism of action	(Anjaneyulu et al., 2003; Clarke and Ramsay, 2011)
		*in vivo*	↓MAO isoenzymes	Yoshino et al. (2011)
			↑ BDNF	(Fang et al., 2020)
			Regulation of Copine 6 and TREM1/2 imbalance	
	Resveratrol	Dose = 20–80 mg/kg mice	↓immobility period in mouse models of behavioral despair without affecting locomotor activity.↑noradrenaline, ↑serotonin	(Yáñez et al., 2006; Xu et al., 2010a)
		*in vivo*	↓MAO isoenzymes	
			↓ serotonin uptake	
	Hesperidin	Dose = 0,1–1 mg/kg mice	↓immobility period and the antidepressant-like activity was independent of alterations in locomotor activity anti-inflammatory	(Raza et al., 2011; Carlos Filho et al., 2013)
		*in vivo*	antioxidant activity	
	Rutin	Dose = 0,1–3 mg/kg mice	↓inactivity in TST modulation of monoaminergic neurotransmitter systems	(Machado et al., 2008; Ramos-Hryb et al., 2018))
		*in vivo*		
	Naringenin	Dose = 0,1–50 mg/kg mice	↓immobility in the TST	(Olsen et al., 2008) (Olsen et al., 2008) (Olsen et al., 2008) (Olsen et al., 2008) (Olsen et al., 2008) (Olsen et al., 2008) (Olsen et al., 2008) (Olsen et al., 2008) (Olsen et al., 2008)
		*in vivo*	↓pro-inflammatory mediators	
	Proanthocyanidins polyphenols	Dose = 25–50 mg/kg mice	↓alterations in the locomotor activity	(Xu et al., 2010b; Wang et al., 2012)
		*in vivo*	↑serotonin	
			↑noradrenaline	
			↑synaptic plasticity	
	Nobiletin	Dose = 25–100 mg/kg mice	↓immobility period in both FST and TST serotoninergic, noradrenergic, dopaminergic effects	Yi et al. (2011)
		*in vivo*		
Tannins	Tannic acid	Dose = 30 mg/kg rats	↑levels of monoaminergic neurotransmitters in the brain	Luduvico et al. (2020)
		*in vivo*	Non-selective inhibitor of monoamine oxidase	
Iridoids	Geniposide	Dose = 25, 50, 100 mg/kg rats	Upregulation the hypothalamic GR_α_ mRNA level	Cai et al. (2015)
		*in vivo*	Upregulation the GR_α_ protein expression	
Coumarins	Scopoletin	Dose = 1–100 mg/kg mice	Activation of postsynaptic α_1_- and α_2_-adrenoceptors	Capra et al. (2010)
		*in vivo*		
	Umbelliferone	Dose = 15 mg/kg, 30 mg/kg rats	Downregulation of Rho-associated protein kinase (ROCK) signaling	Qin et al. (2017)
		*in vivo*	Upregulation of protein kinase B (Akt) signaling	
	*Hypericum perforatum*		Monoamine reuptake inhibitor	Sarris et al. (2021)
			Supportive towards the hypothalamic pituitary adrenal axis	

Symbols: ↑, increase, ↓, decrease.

For example, membrane-like alkaloids in plants like *Narcissus* (Amaryllidaceae) and *Sceletium* have potential antidepressant properties (Hanks, 2002; Berkov et al., 2020). *Narcissus* is a source of neuroactive substances like galantamine that has been used in the treatment of Alzheimer’s disease (Smith et al., 1996). Mesembrine-like alkaloids demonstrated some SSRI activity in mood disorders (Gericke and Van Wyk, 2001a). In addition, mesembrine alkaloids have been shown to phosphodiesterase-4 (PDE-4) inhibition. They act by changing the levels of cyclic AMP (cAMP) as well as the induction of Brain-Derived Neurotrophic Factor (BDNF) mRNA, which has an antidepressant effect in patients who accompany MDD (Fujimaki et al., 2000).

Polyphenols like curcumin (*Curcuma longa*) are strongly recommended in the treatment procedures for MDD (Darvesh et al., 2012) (Table 2). Some authors reported that curcumin affects stressed mice by modulation of the various neurotransmitter systems in forced swim test (FST), similar to imipramine affection (Xu et al., 2005a; Xu et al., 2007). In another study, modulation of the serotoninergic system was approved via the cAMP pathway induced by curcumin (Li et al., 2009). Also, glutamate receptors are involved in curcumin’s antidepressant effect by inhibiting the presynaptic voltage-gated calcium channels (Lin et al., 2011). In one study, the inhibitory effect of curcumin on glutamate release and the enhancement of the antidepressant activity of fluoxetine were reported (Kulkarni et al., 2008; Wang et al., 2008; Wang et al., 2010; Lin et al., 2011; Zhang et al., 2013). In the reports, apigenin, one of the bioflavonoids in behavioral test models, displayed significant anti-immobility action and neurotransmitters turnover induction in the mice model (Nakazawa et al., 2003). Moreover, haloperidol reversed the antidepressant action of apigenin (Han et al., 2007). The molecular mechanism behind its antidepressant activity was the inhibition of interleukin 1β and the activation of NLRP3 inflammasome in rat brains (20 mg/kg b. w., intragastrically) (Li et al., 2016). Amentoflavone is a bioflavonoid apigenin dimer (Hossain et al., 2021; Rajib et al., 2021), inhibited the flumazenil binding to rat brain at GABA receptors (Gutmann et al., 2002; Colovic et al., 2008; Ishola et al., 2012). Some authors reported that oral administration of amentoflavone in forced swim test (FST) was more potent than imipramine (Ishola et al., 2012).

**FIGURE 1:**
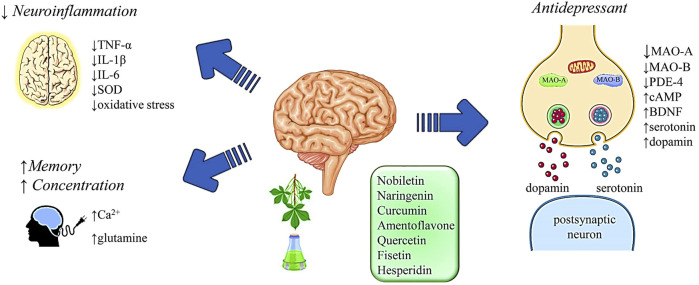
Schematic illustration of the possible mechanisms of natural compounds in neuropsychiatric disorders. Abbreviations and symbols: ↑, increase; ↓, decrease; TNF-α, Ca^2+^ tumour necrosis alpha; IL, interleukin; SOD, superoxide dismutase; MAO, monoaminoxidase; PDE-4, phosphodiesterase 4; cAMP, cyclic adenosine monophosphate; BDNF, brain-derived neurotrophic factor.

In other studies, chlorogenic acid, a polyphenol (in coffee), could enhance mood in patients (Cropley et al., 2012). The mechanism of the antidepressant action of chlorogenic acid was hypothesized to act through the opioidergic pathway (Kwon et al., 2010; Park et al., 2010; Girish et al., 2012), but also reduce neuroinflammation and oxidative stress conditions (Chen et al., 2021). Ferulic acid (FA) induces an anti-immobility effect in behavioral despair models, including FST and TST (Zeni et al., 2012) and can be effectively supplemented in depressive disorders accompanying epilepsy (Singh and Goel, 2016). Some research showed the antidepressant activity of quercetin bioflavonoid by inhibiting MAO activity in the brain (Figure 1) (Butterweck et al., 2000; Haleagrahara et al., 2009; Clarke and Ramsay, 2011; Lam et al., 2012; Soofiyani et al., 2021) and by regulating the copine 6 and TREM1/2 imbalance related to the BDNF factor (Fang et al., 2020). In addition, quercetin showed antidepressant-like action in streptozotocin-induced diabetic mice compared to fluoxetine or imipramine (Kaur et al., 2007; Kawabata et al., 2010). Quercetin in some studies showed the inhibition of the breakdown of serotonin neurotransmitters in mouse brain mitochondria (Yoshino et al., 2011). The other molecule, hesperidin reduced the immobility period in the locomotor activity animal model (Souza et al., 2013).

Other acts of hesperidin are anti-inflammatory (reduction of TNF-α, Interleukin 1 beta (IL-1b) levels) and antioxidant activity in strokes (Figure 1) (Raza et al., 2011). *Hypericum perforatum* has a glycoside flavonol—rutin–that is used for the treatment of depression (Machado et al., 2008; Galeotti, 2017 ) and exhibits anti-inflammatory properties (Parashar et al., 2017) and immobility time-reducing action (30–120 mg/kg p.o. in mice) (Yusha’u et al., 2017). Rutin showed spatial memory enhancement and increased the levels of natural polyphenols in managing significant depression in the hippocampus of aged rat brains (Pyrzanowska et al., 2012). Resveratrol, another phenolic compound in grapes, significantly decreases the immobility period in animal models of locomotor activity and increases noradrenaline and serotonin levels (Yáñez et al., 2006; Xu et al., 2010b; Park et al., 2012; Zhang et al., 2012). The antidepressant action of resveratrol increased dopamine in the brain of female mice, similar to synthetic estrogen (Di Liberto et al., 2012). The antidepressant activity of the anthocyanidins in animal models was indicated by scientists and antidepressant activity in the animal model was due to the change in the locomotor activity (Xu et al., 2010a; Yi et al., 2011).

### 3.2 Schizophrenia

#### 3.2.1 Treatment of schizophrenia using approved drugs

Another mental disorder characterized by periods of continuous or recurrent psychosis with symptoms such as delusions, hallucinations, disorganized speech or behaviour, and impaired cognitive ability is called schizophrenia (World Health Organization, 1992; Lavretsky, 2008). The most important pathophysiological cause of the disease is abnormalities in neurotransmitters such as dopamine, serotonin, glutamate, aspartate, glycine, and gamma-aminobutyric acid (GABA) (Lavretsky, 2008). The prevalence of the disease in the United States is estimated to be between 0.6 and 1.9, and the prevalence is the same in men and women, but the onset of symptoms is seen faster in men than in women (Wu et al., 2006; Van Os and Kapur, 2009).

#### 3.2.2 Treatment of schizophrenia and bioactive compounds

Schizophrenia treatment is divided into two categories: pharmacological and non-pharmacological: non-pharmacological treatments include targeting symptoms, preventing recurrence of the disease, and increasing adaptive function to eventually return the person to the community (Dipiro et al., 2014). The individual, group, and cognitive-behavioural psychotherapeutic therapies can also be used in non-pharmacological treatments (Dickerson and Lehman, 2011). Drug therapies include the use of first-generation antipsychotics, which are dopamine and serotonin antagonists such as lumateperone, risperidone (Marder and Meibach, 1994; Blair, 2020), clozapine (Leponex) (Stahl and Meyer, 2020), olanzapine (Zyprexa) (Bhana et al., 2001), quetiapine (Komossa et al., 2010), and ziprasidone (Lüllmann and Mohr, 2006). Also, fluoxetine was proved to bring positive outcomes when administered to patients, as it induced slight decrease in depressive symptoms (Spina et al., 1994). Some classifications of natural products are determined for their antipsychotic potentials, such as terpenoids, beta-caryophyllene, and limonene. Also, the antipsychotic saponin, polygalasaponin, was recognized for possessing antipsychotic properties by inhibiting cannabinoid receptors (Chung et al., 2002; Ajao et al., 2018). In the study of Abdul Rahim et al. 2022 *Polygonum minus* leaf extract (100 mg/L, 4 days) was found to decrease the level of cortisol in a zebrafish anxiety model, similarly to fluoxetine. In another study, a coumarin–scopoletin was described as an antidopaminergic agent with a U-shaped dose dependent activity towards the stereotyped behaviors in mice. The dose of 0.1 mg/kg b. w. (*per os*) was found effective in the alleviation of positive symptoms of schizophrenia psychosis. Another natural product, the derivative of anthracene–emodin was found to interfere with the schizophrenic responses induced in murine models (Mitra et al., 2018). The attenuation of pre-pulse inhibition and improvement of startle reponses were observed in neonatal rats treated with 15 and 50 mg/kg emodin in a subchronic model. Its possible mechanism of action may be related to the stimulation of the phosphorylation process of both ErbB1 and ErbB2. The efficacy of curcumin was determined in several *in vivo* clinical trials. This phenolic compound from turmeric tuber was administered to 36 schizophrenic patients (360 mg/day for 8 weeks) in a double-blind, placebo-controlled study to research its impact on the BDNF that is engaged in the neurodegeneration and cell survival processes (x). The compound was found to increase the level of BDNF. Furthermore, Hosseininasab and co-investigators (2021) described the influence of curcumin on both positive and negative symptoms in an 8-weeks- long clinical trial with 300 mg of curcumin added to the conventional medication. Curcumin was proved to alleviate memory processes and decrease the IL-6 levels and was well-tolerated by the patients. Table 3 presents natural products and their mechanism of action which were tested in the treatment of schizophrenia.

**TABLE 3:** The most representative bioactive compounds and their major effects in treatment and prevention of schizophrenia.

Disease	Main group of compounds		Neuro-biological functions	References
Schizophrenia	Alkaloids	Huperzine A	reversible AChE inhibitor	(Zangara, 2003; Wang et al., 2006)
		L-SPD	agonist on D_1_ receptors in the medial prefrontal cortex (mPFC)	Mo et al. (2007)
	*Polygonum minus* leaf extract		↓ cortisol level in zebrafish model	(Nurhidayaha et al., 2022)
	Coumarin	Scopoletin	↓positive symptoms and stereotyped behavior	Pandy and Vijeepallam (2017)
			Antidopaminergic activity	
	Anthraquinone	Emodin	↑ phosphorylation process of both ErbB1 and ErbB2	Mitra et al. (2022)
			↓ pre-pulse inhibition and improvement of startle reponses in rats dose = 15–50 mg/kg b.w	
	Phenolic compounds	Curcumin	improvement of positive and negative scales	Hossain et al. (2021)
			↓ IL-6, ↑BDNF	Wynn et al. (2018)
			24-weeks, double-blind, randomized, placebo-controlled study on thirty-eight patients with chronic schizophrenia. 3,000 mg/d curcumin or placebo combined with antipsychotics. significant response to curcumin in the treatment of negative symptoms	Miodownik et al. (2019)

### 3.3 Bipolar disorder

Bipolar disorder or chronic manic depression manifests as a recurrent illness with symptoms of depression or manic (Jann, 2014). The disease most often affects adolescents or adults, and sometimes the elderly (Tiihonen et al., 2017). The disease is classified into two categories: type I (episodes of depression and persistent mania) and type II (episodes of depression and hypomania) (Cooper, 2018). The prevalence of this disease worldwide is 1%–3% and its incidence is the same in men and women considering different ethnicities and races (Ferrari et al., 2011; Moreira et al., 2017). The exact pathophysiology of the disease has not yet been determined, but more than 85% of cases are due to heredity (McGuffin et al., 2003). It has been shown that there is a relative overlap of the catechol-o-methyltransferase (COMT) gene for schizophrenia and bipolar disorder, which controls dopamine metabolism (Berrettini, 2003; Murray et al., 2004).

#### 3.3.1 Treatment of bipolar disorder using approved drugs

To treat Bipolar Disorder, two psychosocial methods (using physical methods to establish individual relationships to help change the behaviour of the individual in society) (Woodward, 2015) and pharmacological therapies are used. Medications include the use of mood stabilizers such as lamotrigine, lithium, clozapine, divalproex, carbamazepine, olanzapine, and atypical antipsychotics such as quetiapine, risperidone, aripiprazole, and ziprasidone; and antidepressants such as bupropion and SSRIs (Jain, 2020). Herbal products can be considered to treat symptoms of insomnia and anxiety in bipolar patients. Valerian, chamomile, ginkgo, hops, and passionflower might be beneficial. However, some of their constituents’ effectiveness and safety have not been approved and need more studies (Baek et al., 2014).

#### 3.3.2 Treatment of bipolar disorder and bioactive compounds

Oxidative stress is one of the major factors described in the etiology of mania. That is why several experimental studies focus on the development of drug candidates that could restore oxidation-reduction balance. In the light of this information, natural products that are proved to exhibit antioxidant properties are important to drug candidates in the reduction of manic episodes (Recart et al., 2021). Herbal intervention in bipolar disorder is recommended and prescribed, accompanied by mood stabilizers (Currier and Trenton, 2002; Mohr et al., 2005). *Hypericum perforatum* might not be used in patients alone. A clinical trial using ashwagandha provided substantial benefits for cognitive performance compared with a placebo (Chengappa et al., 2013). Ethanolic extracts of saffron (*Crocus sativus*) have been used in preclinical animal models, and its constituents, safranal, and crocin have shown antidepressant effects (Hosseinzadeh and Noraei, 2009). *Curcuma longa* (turmeric) and *H. perforatum* (St John’s wort) are other plants used in various nervous system disorders and have been used over the past decades in the treatment of MDD (Gopi et al., 2017; Kunnumakkara et al., 2017). Acute and chronic administration of carvone (50 and 100 mg/kg, i. p.)—a monoterpene present in volatile oils of several plant species, e.g., *Mentha* spp., *Carum carvi*, and others–in a methylphenidate mice mania model resulted in a decreased locomotor activity in the tested animals, possibly thanks to the GABAergic activity and sodium channels blockage (Nogoceke et al., 2016). Gallic acid (GA) a phenolic acid that is widely spread in the plant kingdom was used in the treatment of ketamine-induced mania in rats and compared to the action of lithium. Similarly to lithium (45 mg/g twice a day) GA (50 and 100 mg/kg) administered for 14 days decreased the hyperlocomotion of the animals, induced the antioxidant properties and prevented the cholinergic disfunctions in the brain (Recart et al., 2021). In the studies of Kanazawa and collaborators (2016, 2017) quercetine administered intraperitoneally (10–40 mg/kg b. w.) showed antioxidant properties and inhibition of protein kinase C. In turn the flavonoid regulated sleep deprivation and diminished the induced hyperlocomotion in mice. Table 4 summarizes natural compounds which are used in the treatment of bipolar disorders.

**TABLE 4:** Bioactive compounds and their major effects in the treatment of bipolar disorders.

Disease	Main group of compounds	Neuro-biological functions	References
Bipolar Disorder	Ginkgo	↑cerebrovascular blood flow	Nourbala and Akhoundzadeh, (2006)
		↓hyperactivity	
	Monoterpenes	GABAergic activity	Nogoceke et al. (2016)
	Carvone	↓ locomotor activity sodium channels blockage	
	Phenolic compounds	↓ free radicals formation	Recart et al. (2021)
	Gallic acid	↓ hyperactivity prevented cholinergic dysfunctions	
	Quercetin	↓protein kinase C	(Kanazawa et al. (2016), Kanazawa et al. (2017)
		↓ hyperlocomotion	

### 3.4 Autism spectrum disorders

Autism is a disorder of the nervous system that is associated with poor communication, social interaction, and repetitive behaviours, and usually manifests itself in childhood or adolescence (Landa, 2008; Tuchman et al., 2010; Edition, 2013). Causes of autism include immaturity of brain parts (London, 2007), brain-intestinal axis abnormalities (Wasilewska and Klukowski, 2015; Israelyan and Margolis, 2019), synaptic dysfunction (Levy and Ds, 2009), and mutations in the genes of cellular adhesion proteins involved in the synaptic region (Walsh et al., 2008). The prevalence of this disease is 10–16 per 10,000 people, and boys are more likely to develop autism than girls (Fombonne, 2006; Fombonne, 2009). The rate of disease in the United States is increasing every year (Newschaffer et al., 2007).

#### 3.4.1 Treatment of autism spectrum disorders using approved drugs

The treatment for autism includes two categories: pharmacological and non-pharmacological: non-pharmacological treatments include parent education (Kilpatrick et al., 2001), applied behavioural analysis (ABA) (Cooper et al., 2007), treatment and education of children with autism (Schopler et al., 2010), and cognitive-behavioural therapy (CBT) (Wood et al., 2009; Reaven et al., 2012). Atypical antipsychotic drugs called risperidone and aripiprazole can be used to treat aggressive and self-harming behaviours caused by autism (Leskovec et al., 2008; Rapin and Tuchman, 2008; Ji and Findling, 2015). Fluoxetine and fluvoxamine can be used to reduce ritualistic and repetitive behaviours. Methylphenidate is also used to treat hyperactivity in children with autism (Dubowitz et al., 2008).

#### 3.4.2 Treatment of autism spectrum disorders and bioactive compounds

Luteolin, a natural plant flavonoid, significantly counteracted IL-6 in astrocytes (Gullotta et al., 1985; Zuiki et al., 2017; Deb et al., 2020) and exhibited neuroprotective, anti-inflammatory activities (Bertolino et al., 2017). Luteolin formulation (NeuroProtek®) was prescribed accompanied to the drugs of children with ASD (Theoharides et al., 2012). Thus, luteolin was used for managing autistic behaviour and improvement of social behaviour (Chen et al., 2008; Tsilioni et al., 2015; Xu et al., 2015). Luteolin also inhibited the stimulation of activated T cells and reduced inflammatory molecules (Kritas et al., 2013). Daily intake of green tea extract (*Camellia sinensis*), a polyphenols source, is proved to exhibit health effects (Schimidt et al., 2017). This plant enhanced the locomotion activity in valproate-induced autistic mice (Banji et al., 2011; Takeda et al., 2011; Sundberg and Sahin, 2015; Kumaravel et al., 2017; Urdaneta et al., 2018). Major antioxidant enzymes such as superoxide dismutase were increased by catechin, in autistic children (Rossignol and Frye, 2014). The action of the piperine, a major alkaloid isolated from pepper species, displays considerable anti-oxidative effects and enhancement of memory with the regulation of Ca^2+^ ion entry into the neurons and the presynaptic release of glutamine (Wattanathorn et al., 2008; Fu et al., 2010; Pragnya et al., 2014). Piperine is progressing its future beneficial effects in autistic children (Wattanathorn et al., 2008).

Curcumin in *Curcuma longa* was found for its neuroprotective activities and cellular signalling role in regulating oxidative stress (Salehi et al., 2020). Moreover, curcumin could reduce inflammatory factors in diseases and exhibit antioxidant radical scavenging activities (Salehi et al., 2019a; Quispe et al., 2022). As a potential treatment for autism, Ginkgo Biloba extract was used accompanied by risperidone. The results showed that the treated group indicated fewer adverse effects as compared to the control group (Hasanzadeh et al., 2012). Several studies investigated the role of antioxidants and natural anti-inflammatory products such as curcumin, resveratrol, naringenin, and piperine to reduce the symptoms of autism spectrum disorder (*in vivo* and *in vitro*). In a study, curcumin increased the level of antioxidant enzymes and helped diminish dysfunctions. Curcumin in the dose of 200 mg/kg in autistic rats can attenuate oxidative stress and release tumor necrosis factor (TNF-α). However, exploring their potential clinical effects and drug delivery methods is essential (Fu et al., 2010; Al-Askar et al., 2017). Table 5 summarizes the effects of bioactive compounds as potential agents in the treatment of autism.

**TABLE 5:** Natural products used in the treatment of autism.

Disease	Main group of compounds		Neuro-biological functions	References
Autism	Polyphenols	Luteolin	neuroprotective	Bertolino et al. (2017)
			anti-inflammatory	Kritas et al. (2013)
			↓mast cell-dependent stimulation of activated T cells	
			↓histamine	
			↓leukotrienes	
		*Camellia sinensis*	↑dopamine	Takeda et al. (2011)
			↑serotonin	
		Curcumin	Attenuates oxidative stress	(Fu et al., 2010) (Al-Askar et al., 2017) (Salehi et al., 2020)
			↓ TNF- α	
			↑ neuroprotective properties	

### 3.5 Attention deficit hyperactivity disorder

Attention deficit hyperactivity disorder (ADHD) is a mental-behavioural disorder associated with the development of the nervous system that presents with symptoms such as inattention, excessive energy, hyper-fixation, and impulsivity (American Psychiatric Association, 1980; Cotterill, 2019). These people have difficulty controlling their emotions and have difficulty in executive activities (Mandah and Osuagwu, 2020). The exact cause of the disease is not yet fully understood, but in more than 75% of cases, genetic causes are involved (Mandah and Osuagwu, 2020). Also, dysfunction of neurotransmitters such as dopamine and norepinephrine (Chandler et al., 2014; Stansfield, 2019) and signs of signal change in the Central Nervous System (CNS) such as paradoxical reaction is observed in this regard (Langguth et al., 2011). It affects 6%–7% of people in the age group of 18 years (Willcutt, 2012) and the incidence of the disease in men is three times higher than in women (Singh, 2008).

#### 3.5.1 Treatment of attention deficit hyperactivity disorder using approved drugs

Treatments for this disease include behavioural therapies such as psychoeducational input, behaviour therapy, cognitive behavioural therapy, interpersonal psychotherapy, family therapy, school-based interventions, social skills training, behavioural peer intervention, organization training, and parent management training (Health, 2009; Evans et al., 2018; Lopez et al., 2018); Medical counselling; Medications such as stimulants, atomoxetine, alpha-2 adrenergic receptor agonists, and sometimes antidepressants (Wilens and Spencer, 2010; Bidwell et al., 2011); or as a combination therapy. Some studies have recommended the use of methylphenidate (Storebø et al., 2015).

#### 3.5.2 Treatment of attention deficit hyperactivity disorder and bioactive compounds

Natural products, which may be potentially used in the treatment of ADHD were presented in Table 6. American ginseng (*Panax quinquefolium*) in children with ADHD improved significantly on a social problems measure (Lyon et al., 2001; Trebatická et al., 2006). Another plant*, Ginkgo biloba* enhanced cerebrovascular blood flow and reduced hyperactivity due to the lack of focus (Nourbala and Akhoundzadeh, 2006). It has been documented that *Passiflora* might be a novel therapeutic agent for treating ADHD (Salehi et al., 2010; Uebel-von Sandersleben et al., 2014). One study in adults with ADHD revealed that lobeline as an alkaloid improves working memory in patients with no significant impact on the attention noted (Martin et al., 2018). Whereas, a comprehensive study is needed to make more definitive statements regarding the effect of lobeline and the usage of methylphenidate. Lobeline could have different effects based on individual differences. Some pediatric patients with ADHD use natural products such as flavonoids. Although herbal remedies are generally considered safe when used appropriately with other treatment strategies (Martin et al., 2018).

**TABLE 6:** Bioactive compounds and their mechanism of action used as potential drugs in the treatment of ADHD.

Disease	Main group of compounds	Neuro-biological functions	Refs
ADHD	Ginkgo	↑cerebrovascular blood flow	Nourbala and Akhoundzadeh, (2006)
		↓hyperactivity due to boredom and lack of focus	
	*Panax quinquefolium*	Improvement of social problems measure	(Lyon et al., 2001) (Trebatická et al., 2006)
	Lobeline	↑ memory capacities	Martin et al. (2018)
	*Bacopa monnieri*	↓inattention	Kean et al. (2022)
		↓ error-making	
		↓ hyperactivity	
	Pine bark extract	↓inattention	Hsu et al. (2021)
		↓ hypersensitivity	
		↓ hyperactivity	

A double-blind and placebo-controlled randomized trial (112 males aged 6–14 years) in a population of males supplemented with *Bacopa monnieri* extract showed the reduction of hyperactivity, inattention and decreased error-making (Kean et al., 2022). Another clinical trial performed in a group of twenty males and females aged 10 ± 2.1 years described by Hsu and co-investigators (2021) denotes that the administration of 25 or 50 mg pine bark extract for 14 days resulted in a significant reduction of in inattention, hyperactivity, and impulsivity.

### 3.6 Psychiatric disorders associated with epilepsy

Epilepsy is a neurological diseases manifested by recurrent seizures is called epilepsy, which is classified as short and short periods to long and severe periods (Sharifi-Rad et al., 2021b; Kwon et al., 2022). The main mechanisms of epilepsy include abnormal activity in the cerebral cortex, brain damage, stroke, brain tumours, various brain infections, and genetic defects at birth (Begley et al., 2022; Kanner and Bicchi, 2022). The prevalence of this disease varies in different countries and is generally 7.6 people per 1,000 people (Kelvin et al., 2007; Fiest et al., 2017). The incidence of epilepsy is higher in men than in women and affects very young and very old people (Fiest et al., 2017).

#### 3.6.1 Treatment of epilepsy using approved drugs

There are many treatments for epilepsy, including surgery (such as cutting the hippocampus, removing tumors, and removing part of the neocortex) (Ryvlin et al., 2014), specific diet (for instance ketogenic diet) (Martin-McGill et al., 2020), avoidance therapy (reducing or eliminating certain triggers factors such as excessive light) (Verrotti et al., 2005), exercise (Arida et al., 2009), and medication such as midazolam, diazepam (Uk, 2012), lorazepam, phenytoin, lamotrigine, levetiracetam (Uk, 2012), carbamazepine, and valproate, etc. (Nevitt et al., 2018; Nevitt et al., 2019). In Table 2 are summarized data regarding used current pharmacological therapies.

#### 3.6.2 Treatment of epilepsy and bioactive compounds

Lycopene, a carotenoid antioxidant, has neuroprotective properties against oxidative stress and mitochondrial dysfunction in PTZ-induced seizures of epilepsy (Sakurada et al., 2009; Bhardwaj and Kumar, 2016) (Table 7. Some authors reported that the extract of *Nardostachys jatamansi* (Valerianaceae) and the synergistic use with phenytoin reduced mental weakness as well as enhanced the seizure threshold in the animal model of generalized tonic-clonic seizures (Luszczki et al., 2009; Jiang et al., 2015). Aconitum alkaloids induce their anticonvulsant activities via interaction with voltage-dependent Na^+^ channels in various experimental models, including PTZ (Charveron et al., 1984; Chen et al., 1996; Lin et al., 2002; Da Silva et al., 2006; Felipe et al., 2007; Da cruz et al., 2013) (Table 7).

**TABLE 7:** Phytochemicals and their potential effects in treatment and prevention of neuropsychiatric disorders in epilepsy.

Compounds	Main group of compounds	Verified effective concentrations/model	Potential effects	References
Alkaloids	Aconitum	IC_50_ = 0,1–1 µM rats hippocampal slices	↓GABA	Ameri et al. (1996)
	*in vitro*		↓epileptiform activity	
Isoquinoline alkaloids	Montanine	Dose = 64.7–67.6 mg/kg rats	modulation of benzodiazepine GABA_A_ receptors	Da Silva et al. (2006)
		*in vivo*		
	Berberine	Dose = 10–20 mg/kg/i.p. mice	modulation of neurotransmitter systems	Bhutada et al. (2010)
		*in vivo*		
	Tetrahydropalmatine	Dose = 10–30 mg/kg/i.p. mice	↓dopamine output	Lin et al. (2002)
		*in vivo*	↑ cholinergic receptor function	
	Palmatine	Dose = 450 μM/7 days	↓ locomotor activity	Gawel et al. (2020)
		Zebrafish	↓ BDNF and c-fos levels	
		*in vivo*	↓ number and mean duration of events	
Amide alkaloid	Piplartine	Dose = 50–100 mg/kg/i.p. mice	↓epileptiform activity	Felipe et al. (2007)
		*in vivo*		
Ergot alkaloids	no data	different doses	effects at dopaminergic and serotoninergic synapses	Anlezark and Meldrum, (1978)
		*in vivo* and *in vitro*		
Piperidine alkaloids	piperine	Dose = 1–2.5 mg/kg/i.p. mice	modulation of the GABAergic system	Da cruz et al. (2013)
		*in vivo*		
Flavonoids	Hesperidin	Dose = 500 mg/kg mice	↓convulsant effects of PTZ	(Dimpfel, 2006; Kumar et al., 2014)
		*in vivo*	↓effects of enhanced calcium	
	Apigenin	Dose = 25–50 mg/kg rats	↓GABA-activated chloride ion channel	Avallone et al. (2000)
		*in vivo*	GABA antagonist	
			↑effect of diazepam of GABA receptors	
	Fisetin	Dose = 10–25 mg/kg mice	antioxidant	Raygude et al. (2012)
		*in vivo*	↓oxidative damage modulating GABAergic transmission	Liu et al. (2012)
	Wogonin	Dose = 5–10 mg/kg rats	↑ Cl^−^ influx	Park et al. (2007)
		*in vivo*	↓ GABA	
	Baicalein	Dose = 100 mg/kg rats and mice	↑Cl^−^ influx antioxidant	(Yoon et al., 2011; Liu et al., 2012)
		*in vivo*		
	Chrysin	Dose = 3 mg/kg rats and mice	Acting on central BZD receptors	Medina et al. (1990)
		*in vivo*		
	Oroxylin A	Dose = 3.67–60 mg/kg rats	antagonistic effects by adverse action on α-2,3,5 subunits of the GABA receptor	Huen et al. (2003)
		*in vivo*		
	Luteolin	Dose = 10 mg/kg rats	↓frequency of seizures	Birman et al. (2012)
		*in vivo*		
	Hispidulin	Dose = 10 mg/kg rats	positive modulator of GABA receptors	(Kavvadias et al., 2004; Lin et al., 2012)
		*in vivo*	↓voltage-dependent Ca^2+^ entry directly interfering with the exocytotic	
	Naringenin	Dose = 20–40 mg/kg rats	modulation of the benzodiazepine site of the GABA receptors	(Golechha et al., 2014; Shakeel et al., 2017)
		*in vivo*	↓lipid peroxidation	
			↓seizures	
	Rutin	Dose = 90 mg/kg, i.p. rats	Interacting with GABAAbenzodiazepine receptor	Nassiri-asl et al. (2008)
		*in vivo*		
	Vitexin	Dose = 90 mg ⁄kg, i.p. rats	↑GABA	Abbasi et al. (2012)
		*in vivo*	↓oxidative injury	
			Terpenoids	
	α-Terpineol	Dose = 100, 200,400 mg/kg rats	Protective effects against PTZ- and MES-induced convulsive seizures in mice	(De Sousa et al., 2007; Silva et al., 2009)
		*in vivo*		
	Carvacrol borneol	Dose = 50, 100, 200 mg/kg mice	↓GABA	Quintans-Júnior et al. (2010)
		*in vivo*		
	Isopulegol	Dose = 200 mg/kg rats	Positive modulation of benzodiazepine sensitive	Silva et al. (2009)
		*in vivo*	GABA receptors antioxidant	
	Eugenol	Dose = 100 mg/kg rats	↓neuronal excitability	Huang et al. (2012)
		*in vivo*	↑Ina inactivation	
			↓INa (NI)	
	Ursolic acid	Dose = 2.3 mg/kg rats and mice	↓GABA	(Taviano et al., 2007; Kazmi et al., 2012)
		*in vivo*		
Saponins	Saikosaponin	IC_50=_1 µM *in vitro*	Voltage-gated sodium channel blocking	(Yu et al., 2012; Zhu et al., 2014)
	saponins fractions	Dose = 1, 2, 4 mg/kg mice	↓GABA	Singh and Goel, (2016)
		*in vivo*	↓calcium and sodium channel functions	
Phenolic compounds	6-gingerol	Dose=37.5 μM/6 days	↓GLU level	(Gawel et al., 2021)
		Zebrafish	↓GLU/GABA ratio	
		*in vivo*	↓ frequency of seizures	
			↓ length of seizures	
Coumarins	Esculetin	Dose = 1, 2, 5 mg/kg mice	↓seizures	Woo et al. (2011)
		*in vivo*	↓GABA	
	Osthole	Dose = 259–631 mg/kg mice	GABA modulation	(Luszczki et al., 2009; Łuszczki et al., 2010; Zhu et al., 2014)
		*in vivo*		
	Imperatorin	Dose = 300 mg/kg mice		
		*in vivo*		
	Oxypeucedanin	Dose = 300 mg/kg mice		
		*in vivo*		

Many flavonoids like hesperidin that prevent tonic-clonic seizures increased the protective effect of N-nitro-L-arginine methyl ester (L-NAME) on kindling induced by pentylenetetrazole (PTZ) as well as enhanced diazepam’s effect. Phytochemicals and their biological function in the treatment of mentioned neuropsychiatric diseases except psychiatric disorders associated with epilepsy are summarized in Table 7 (Fernández et al., 2005; Kumar et al., 2013; Kumar et al., 2014). Apigenin acts as a GABA antagonist at flumazenil-insensitive α_1_β_2_ GABA receptors (Avallone et al., 2000). In addition, naringin has an anticonvulsant effect in kainic acid and PTZ models (Golechha et al., 2011; Golechha et al., 2014; Jeong et al., 2015). An alkaloid, piperine, has been recognized as an adjunct therapy with antiepileptic drugs, carbamazepine, and phenytoin. Administration of piperine could increase the bioavailability of synthetic anti-epilepsy drugs and decrease the adverse effects of synthetic drugs by diminishing the dose. On the other hand, apigenin, a flavonoid, can decrease the myeloperoxidase-mediated oxidative stress and inhibit cell death dependent on iron. It is characterized by the accumulation of lipid peroxides (ferroptosis) for rapidly discovering additional antiepileptic agents to prevent and treat epilepsy. Moreover, apigenin and other flavonoids have potentially antiepileptic and neuroprotective activity by inhibiting the glutamate receptors in mice (Aseervatham et al., 2016; Shao et al., 2020).

Zebrafish model was found to be an efficient screening method for the development of new drug candidates with antiseizure properties. In the studies of Gawel and co-investigators, palmatine from *Beberis sibirica* and 6-gingerol isolated from *Zingiber officinale* were effectively reducing the length of seizures and their number. The effect of 6-gingerol administration might have been achieved by the reduced glutamate and glutamate-to-GABA ratio levels in the fish brains analyzed by HPLC-MS instrumentation (Gawel et al., 2021). The administration of palmatine (450 μM, 7 days) decreased *c-fos* and BDNF levels, whereas, in the behavioral assay, palmatine decreased locomotor activity of animals. The described activity was higher in the combination with berberine (Gawel et al., 2020).

## 4 Limitations, challenges and clinical gaps

Psychiatric disorders are mental health problems characterized by different symptoms. The classification of mood disorders is still ambiguous. Some categories are defined as subgroups due to the symptoms (Enatescu et al., 2020; Trofor et al., 2020). The cause of these disorders is social, environmental, genetic issues, or psychotropic drugs. Neurological and psychiatric disorders account for 13% of the world’s total complications (Mondiale de la Santé, 2013). Many natural remedies are alternative procedures to increase the effectiveness of prescription drugs (Akhondzadeh, 2007; Salehi et al., 2019b; Sharifi-rad et al., 2021a). Herbal medicines contain a wide range of medicinal compounds with therapeutic effects (Butnariu et al., 2022; Taheri et al., 2022). Nowadays, many synthetic drugs originated from herbal medicines (Sharifi-rad et al., 2021d; Alshehri et al., 2022). Herbal medicines are still used in many diseases, primarily mental and neurological disorders (Sharifi-Rad et al., 2021c; Tsoukalas et al., 2021). According to the group of authors, plants used in traditional medicine contain main groups of components (Hossain et al., 2022; Painuli et al., 2022; Sharifi-Rad et al., 2022). Tropane alkaloids (antagonists of acetylcholine) known as atropine, scopolamine, and hyoscyamine isolated from *Datura* sp*.* have some anticholinergic activities (Taïwe and Kuete, 2014). For instance, scopolamine is an anti-muscarinic used as a sedative and analgesic (Steenkamp et al., 2004). The anti-muscarinic and anticholinergic effects of these compounds may explain the use of *Datura* in treating mental illness (Maiga et al., 2005). Anxiety effects and neuroprotective activity have been reported in flavonoids. They can bind to GABA receptors with significant affinity (Zhang, 2004). Quercetin significantly reduces ischemic brain damage (Lake, 2000; Dajas et al., 2003; Guenne et al., 2016).

The therapeutic limitations of these compounds are represented by cytotoxic and cardiotoxic effects and must be used with caution (Al-snafi, 2015). For example, securinin acts like strychnine in the range of 5–30 g/kg and causes spasms and death due to respiratory arrest (Maiga et al., 2005). Therefore, controlled use of these herbs should be promoted.

Integrative medicine concerning mental health is a concept that has developed a lot lately, in the conditions in which psychiatry no longer communicates notable advances in psychopharmacology in recent years. In this conjuncture of relative pharmacological stagnation, the complementary natural therapies capture the psychiatric patient, to the detriment of the indications from the treatment guidelines accepted by the psychiatric specialists. But extensive research to explore the combination of bioactive natural componds with synthetic psychotropic drugs in the treatment of mental disorders is needed in the future.

The limitations of the current review are the inclusion in the study of evidence from preclinical pharmacological models, and meta-analyzes focused on the therapeutic impact of bioactive compounds in psychiatric diseases and not from individual clinical trials. On the other hand, the inclusion and analysis of these meta-analyzes is a strong point of this review, as they focused on potential pharmacological mechanisms of action, thus opening new therapeutic windows beneficial to natural bioactive compounds in the therapy of neuropsychiatric diseases.

Although comparative studies have been scrutinized in the pre-clinical area, no clinical trial has been found where herbal medicines are compared to drugs approved by the FDA for the treatment of psychiatric disorders. This is very important to highlight because it must be clear that evidence for the clinical efficacy of these products is not confirmed by head-to-head comparative studies and the conclusions concerning their efficacy derive only from preclinical experimental studies.

## 5 Overall conclusion

There are many factors behind the growing popularity of herbal remedies for a variety of chronic diseases. Many people who use herbal remedies know that health care alternatives are more in line with their values, beliefs, and philosophical orientations towards health and life. Although many chemical drugs are available to treat mental disorders, clinicians have found that many patients are unable to tolerate the side effects of chemical drugs or do not respond well enough. Many herbal remedies have far fewer side effects. Therefore, they can be used as an alternative treatment and could increase the effectiveness of prescription drugs. While the demand for herbal medicines is increasing, herbal extracts and active ingredients isolated from them need to be scientifically approved before being widely accepted and used. Therefore, “phytochemicals” may guarantee a new source of beneficial neuroleptics.

## Abbreviations

ABA, applied behavioural analysis; b.w., body weight; BDNF, brain-derived neurotrophic factor; COMT, catechol-O-methyltransferase; CNS, central nervous system; CBT, cognitive-behavioural therapy; cAMP, cyclic adenosine monophosphate; FA, ferulic acid; GABA, gamma-aminobutyric acid; HPA, hypothalamic-pituitary-adrenal; IL, interleukin; IL-1β, interleukin 1 beta; i.p., intraperitoneal administration; MDD, major depressive disorder; MAOIs, monoamine oxidase inhibitors; p.o., oral administration; PDE-4, phosphodiesterase-4; SNRIs, serotonin-norepinephrine reuptake inhibitors; SOD, superoxide dismutase; TNF-α, tumor necrosis factor-alpha.

## References

[B1] Abbasi E. Nassiriasl M. Shafeei M. Sheikhi M. (2012). Neuroprotective effects of vitexin, a flavonoid, on pentylenetetrazole‐induced seizure in rats. Chem. Biol. Drug Des. 80, 274–278. 10.1111/j.1747-0285.2012.01400.x 22554436

[B318] Abdul Rahim N. Nordin N. Ahmad Rasedi N. I. S. Mohd Kauli F. S. Wan Ibrahim W. N. Zakaria F. (2022). Behavioral and cortisol analysis of the anti-stress effect of *Polygonum minus* (Huds) extracts in chronic unpredictable stress (CUS) zebrafish model. Comp. Biochem. Physiol. Part C: Toxicol. Pharmacol. 256, 109303. 10.1016/j.cbpc.2022.109303 35202824

[B2] Ajao A. A.-N. Alimi A. A. Olatunji O. A. Balogun F. O. Saheed S. A. (2018). A synopsis of anti-psychotic medicinal plants in Nigeria. Trans. R. Soc. S. Afr. 73, 33–41. 10.1080/0035919x.2017.1386138

[B3] Akhondzadeh S. (2007). “Herbal medicines in the treatment of psychiatric and neurological disorders,” in Low-cost approaches to promote physical and mental health (Springer).

[B4] Al-askar M. Bhat R. S. Selim M. AL-Ayadhi L. EL-Ansary A. (2017). Postnatal treatment using curcumin supplements to amend the damage in VPA-induced rodent models of autism. BMC Complement. Altern. Med. 17, 259–311. 10.1186/s12906-017-1763-7 28486989 PMC5424332

[B5] Al-snafi A. E. (2015). The chemical constituents and pharmacological importance of Chrozophora tinctoria. Int J Pharm Rev Res 5, 391–396.

[B6] Allen M. H. Hirschfeld R. M. Wozniak P. J. Baker P. D. Jeffrey D. Bowden C. L. (2006). Linear relationship of valproate serum concentration to response and optimal serum levels for acute mania. Am. J. Psychiatry 163, 272–275. 10.1176/appi.ajp.163.2.272 16449481

[B7] Alshehri M. M. Quispe C. Herrera-Bravo J. Sharifi-Rad J. Tutuncu S. Aydar E. F. (2022). A review of recent studies on the antioxidant and anti-infectious properties of *Senna* plants. Oxid. Med. Cell. Longev. 2022, 6025900. 10.1155/2022/6025900 35154569 PMC8837466

[B8] Ameri A. Gleitz J. Peters T. (1996). Aconitine inhibits epileptiform activity in rat hippocampal slices. Naunyn. Schmiedeb. Arch. Pharmacol. 354, 80–85. 10.1007/BF00168710 8832592

[B9] American Psychiatric Association A. (1980). Diagnostic and statistical manual of mental disorders. Washington, DC: American Psychiatric Association.

[B10] Anjaneyulu M. Chopra K. Kaur I. (2003). Antidepressant activity of quercetin, a bioflavonoid, in streptozotocin-induced diabetic mice. J. Med. Food 6, 391–395. 10.1089/109662003772519976 14977450

[B11] Anlezark G. Meldrum B. (1978). Blockade of photically induced epilepsy by ‘dopamine agonist’ergot alkaloids. Psychopharmacology 57, 57–62. 10.1007/BF00426958 96470

[B12] Arida R. M. Scorza F. A. Scorza C. A. Cavalheiro E. A. (2009). Is physical activity beneficial for recovery in temporal lobe epilepsy? Evidences from animal studies. Neurosci. Biobehav. Rev. 33, 422–431. 10.1016/j.neubiorev.2008.11.002 19059282

[B13] Aseervatham G. S. B. Suryakala U. Sundaram S. Bose P. C. Sivasudha T. (2016). Expression pattern of NMDA receptors reveals antiepileptic potential of apigenin 8-C-glucoside and chlorogenic acid in pilocarpine induced epileptic mice. Biomed. Pharmacother. 82, 54–64. 10.1016/j.biopha.2016.04.066 27470339

[B14] Association A. P. (2006). American psychiatric association practice guidelines for the treatment of psychiatric disorders: Compendium 2006. American Psychiatric Pub.

[B15] Atkinson J. H. Slater M. A. Williams R. A. Zisook S. Patterson T. L. Grant I. (1998). A placebo-controlled randomized clinical trial of nortriptyline for chronic low back pain. pain 76, 287–296. 10.1016/S0304-3959(98)00064-5 9718247

[B16] Avallone R. Zanoli P. Puia G. Kleinschnitz M. Schreier P. Baraldi M. (2000). Pharmacological profile of apigenin, a flavonoid isolated from *Matricaria chamomilla* . Biochem. Pharmacol. 59, 1387–1394. 10.1016/s0006-2952(00)00264-1 10751547

[B17] Baek J. H. Nierenberg A. A. Kinrys G. (2014). Clinical applications of herbal medicines for anxiety and insomnia; targeting patients with bipolar disorder. Aust. N. Z. J. Psychiatry 48, 705–715. 10.1177/0004867414539198 24947278

[B18] Banaschewski T. Roessner V. Dittmann R. W. Santosh P. J. Rothenberger A. (2004). Non–stimulant medications in the treatment of ADHD. Eur. Child. Adolesc. Psychiatry 13, i102–i116. 10.1007/s00787-004-1010-x 15322961

[B19] Banji D. Banji O. J. Abbagoni S. Hayath M. S. Kambam S. Chiluka V. L. (2011). Amelioration of behavioral aberrations and oxidative markers by green tea extract in valproate induced autism in animals. Brain Res. 1410, 141–151. 10.1016/j.brainres.2011.06.063 21820650

[B20] Baureithel K. H. Büter K. B. Engesser A. Burkard W. Schaffner W. (1997). Inhibition of benzodiazepine binding *in vitro* by amentoflavone, a constituent of various species of Hypericum. Pharm. Acta Helv. 72, 153–157. 10.1016/s0031-6865(97)00002-2 9204773

[B21] Begley C. Wagner R. G. Abraham A. Beghi E. Newton C. Kwon C. S. (2022). The global cost of epilepsy: A systematic review and extrapolation. Epilepsia 63, 892–903. 10.1111/epi.17165 35195894

[B22] Berkov S. Osorio E. Viladomat F. Bastida J. (2020). Chemodiversity, chemotaxonomy and chemoecology of Amaryllidaceae alkaloids. Alkaloids. Chem. Biol. 83, 113–185. 10.1016/bs.alkal.2019.10.002 32098649

[B23] Berrettini W. (2003). “Evidence for shared susceptibility in bipolar disorder and schizophrenia,” in American journal of medical genetics Part C: Seminars in medical genetics (Wiley Online Library), 59–64. 10.1002/ajmg.c.20014 14601037

[B24] Bertolino B. Crupi R. Impellizzeri D. Bruschetta G. Cordaro M. Siracusa R. (2017). Beneficial effects of co‐ultramicronized palmitoylethanolamide/luteolin in a mouse model of autism and in a case report of autism. CNS Neurosci. Ther. 23, 87–98. 10.1111/cns.12648 27701827 PMC6492645

[B25] Bhana N. Foster R. H. Olney R. Plosker G. L. (2001). Olanzapine: An updated review of its use in the management of schizophrenia. Olanzapine. Drugs 61, 111–161. 10.2165/00003495-200161010-00011 11217867

[B26] Bhardwaj M. Kumar A. (2016). Neuroprotective effect of lycopene against PTZ‐induced kindling seizures in mice: Possible behavioural, biochemical and mitochondrial dysfunction. Phytother. Res. 30, 306–313. 10.1002/ptr.5533 26633078

[B27] Bhutada P. Mundhada Y. Bansod K. Dixit P. Umathe S. Mundhada D. (2010). Anticonvulsant activity of berberine, an isoquinoline alkaloid in mice. Epilepsy Behav. 18, 207–210. 10.1016/j.yebeh.2010.03.007 20638957

[B28] Bidwell L. C. Mcclernon F. J. Kollins S. H. (2011). Cognitive enhancers for the treatment of ADHD. Pharmacol. Biochem. Behav. 99, 262–274. 10.1016/j.pbb.2011.05.002 21596055 PMC3353150

[B29] Birman H. Üzüm G. Dar K. A. Kapucu A. Acar S. (2012). Effects of luteolin on liver, kidney and brain in pentylentetrazol-induced seizures: Involvement of metalloproteinases and NOS activities. Balk. Med. J. 29, 188–196. 10.5152/balkanmedj.2011.030 PMC4115855 25206993

[B30] Blair H. A. (2020). Lumateperone: First approval. Drugs 80, 417–423. 10.1007/s40265-020-01271-6 32060882

[B31] Brenner G. M. Stevens C. (2017). Brenner and stevens’ pharmacology E-book. Elsevier Health Sciences.

[B32] Burris K. D. Molski T. F. Xu C. Ryan E. Tottori K. Kikuchi T. (2002). Aripiprazole, a novel antipsychotic, is a high-affinity partial agonist at human dopamine D2 receptors. J. Pharmacol. Exp. Ther. 302, 381–389. 10.1124/jpet.102.033175 12065741

[B33] Butnariu M. Quispe C. Herrera-Bravo J. Sharifi-Rad J. Singh L. Aborehab N. M. (2022). The pharmacological activities of *Crocus sativus* L.: A review based on the mechanisms and therapeutic opportunities of its phytoconstituents. Oxid. Med. Cell. Longev. 2022, 8214821. 10.1155/2022/8214821 35198096 PMC8860555

[B34] Butterweck V. Jürgenliemk G. Nahrstedt A. Winterhoff H. (2000). Flavonoids from *Hypericum perforatum* show antidepressant activity in the forced swimming test. Planta Med. 66, 3–6. 10.1055/s-2000-11119 10705724

[B35] Cai L. Li R. Tang W.-J. Meng G. Hu X.-Y. Wu T.-N. (2015). Antidepressant-like effect of geniposide on chronic unpredictable mild stress-induced depressive rats by regulating the hypothalamus–pituitary–adrenal axis. Eur. Neuropsychopharmacol. 25, 1332–1341. 10.1016/j.euroneuro.2015.04.009 25914157

[B36] Cannon J. R. Greenamyre J. T. (2011). The role of environmental exposures in neurodegeneration and neurodegenerative diseases. Toxicol. Sci. 124, 225–250. 10.1093/toxsci/kfr239 21914720 PMC3216414

[B220] Capra J. C. Cunha M. P. Machado D. G. Zomkowski A. D. Mendes B. G. Santos A. R. (2010). Antidepressant-like effect of scopoletin, a coumarin isolated from *Polygala sabulosa* (polygalaceae) in mice: Evidence for the involvement of monoaminergic systems. Eur. J. Pharmacol. 643, 232–238. 10.1016/j.ejphar.2010.06.043 20599906

[B37] Carlos filho B. Del Fabbro L. DE Gomes M. G. Goes A. T. Souza L. C. Boeira S. P. (2013). Kappa-opioid receptors mediate the antidepressant-like activity of hesperidin in the mouse forced swimming test. Eur. J. Pharmacol. 698, 286–291. 10.1016/j.ejphar.2012.11.003 23178563

[B38] Chandler D. J. Waterhouse B. D. Gao W.-J. (2014). New perspectives on catecholaminergic regulation of executive circuits: Evidence for independent modulation of prefrontal functions by midbrain dopaminergic and noradrenergic neurons. Front. Neural Circuits 8, 53. 10.3389/fncir.2014.00053 24904299 PMC4033238

[B39] Charney D. S. Manji H. K. (2004). Life stress, genes, and depression: Multiple pathways lead to increased risk and new opportunities for intervention. Sci. STKE, re5. 10.1126/stke.2252004re5 15039492

[B40] Charveron M. Assié M.-B. Stenger A. Briley M. (1984). Benzodiazepine agonist-type activity of raubasine, a rauwolfia serpentina alkaloid. Eur. J. Pharmacol. 106, 313–317. 10.1016/0014-2999(84)90718-0 6099274

[B41] Chen H.-Q. Jin Z.-Y. Wang X.-J. Xu X.-M. Deng L. Zhao J.-W. (2008). Luteolin protects dopaminergic neurons from inflammation-induced injury through inhibition of microglial activation. Neurosci. Lett. 448, 175–179. 10.1016/j.neulet.2008.10.046 18952146

[B42] Chen K. Kokate T. G. Donevan S. D. Carroll F. I. Rogawski M. A. (1996). Ibogaine block of the NMDA receptor: *In vitro* and *in vivo* studies. Neuropharmacology 35, 423–431. 10.1016/0028-3908(96)84107-4 8793904

[B316] Chen X. D. Tang J. J. Feng S. Huang H. Lu F. N. Lu X. M. Wang Y. T. (2021). Chlorogenic acid improves PTSD-like symptoms and associated mechanisms. Curr. Neuropharmacol. 19 (12), 2180–2187. 10.2174/1570159X19666210111155110 33430733 PMC9185768

[B43] Chengappa K. R. Bowie C. R. Schlicht P. J. Fleet D. Brar J. S. Jindal R. (2013). Randomized placebo-controlled adjunctive study of an extract of *Withania somnifera* for cognitive dysfunction in bipolar disorder. J. Clin. Psychiatry 74, 1076–1083. 10.4088/JCP.13m08413 24330893

[B44] Chiu S. Gericke N. Farina-Woodbury M. Badmaev V. Raheb H. Terpstra K. (2014). Proof-of-concept randomized controlled study of cognition effects of the proprietary extract *Sceletium tortuosum* (zembrin) targeting phosphodiesterase-4 in cognitively healthy subjects: Implications for Alzheimer’s dementia. Evidence-Based Complementary Altern. Med., 1–9. 10.1155/2014/682014 PMC4217361 25389443

[B45] Chiu S. Raheb H. Terpstra K. Vaughan J. Carrie A. (2017). Exploring standardized Zembrin® extracts from the South African plant *Sceletium tortuosum* in dual targeting phosphodiesterase-4 (PDE-4) and serotonin reuptake inhibition as potential treatment in schizophrenia. Int. J. Complement. Altern. Med. 6, 00203. 10.15406/ijcam.2017.06.00203

[B46] Chung I.-W. Moore N. A. Oh W.-K. O'Neill M. F. Ahn J.-S. Park J.-B. (2002). Behavioural pharmacology of polygalasaponins indicates potential antipsychotic efficacy. Pharmacol. Biochem. Behav. 71, 191–195. 10.1016/s0091-3057(01)00648-7 11812522

[B47] Clarke S. E. D. Ramsay R. R. (2011). Dietary inhibitors of monoamine oxidase A. J. Neural Transm. (Vienna). 118, 1031–1041. 10.1007/s00702-010-0537-x 21190052

[B48] Colovic M. Fracasso C. Caccia S. (2008). Brain-to-plasma distribution ratio of the biflavone amentoflavone in the mouse. Drug Metab. Lett. 2, 90–94. 10.2174/187231208784040988 19356077

[B49] Cooper J. O. Heron T. E. Heward W. L. (2007). Applied behavior analysis. 10.1007/BF03392542 PMC2733421 22478071

[B50] Cooper R. (2018). Diagnostic and statistical manual of mental disorders (DSM), 44. United Kingdom of Great Britain and Northern Ireland: KO KNOWLEDGE ORGANIZATION, 668–676.

[B51] Cotterill T. (2019). Principles and practices of working with pupils with special educational needs and disability: A student guide. London: Routledge.

[B52] Cropley V. Croft R. Silber B. Neale C. Scholey A. Stough C. (2012). Does coffee enriched with chlorogenic acids improve mood and cognition after acute administration in healthy elderly? A pilot study. Psychopharmacology 219, 737–749. 10.1007/s00213-011-2395-0 21773723

[B53] Currier G. W. Trenton A. (2002). Pharmacological treatment of psychotic agitation. CNS drugs 16, 219–228. 10.2165/00023210-200216040-00002 11945106

[B54] Da cruz G. M. P. Felipe C. F. B. Scorza F. A. Da Costa M. A. C. Tavares A. F. Menezes M. L. F. (2013). Piperine decreases pilocarpine-induced convulsions by GABAergic mechanisms. Pharmacol. Biochem. Behav. 104, 144–153. 10.1016/j.pbb.2013.01.002 23313550

[B55] Da silva A. F. S. DE Andrade J. P. Bevilaqua L. R. DE Souza M. M. Izquierdo I. Henriques A. T. (2006). Anxiolytic-antidepressant-and anticonvulsant-like effects of the alkaloid montanine isolated from *Hippeastrum vittatum* . Pharmacol. Biochem. Behav. 85, 148–154. 10.1016/j.pbb.2006.07.027 16950504

[B56] Dajas F. Rivera F. Blasina F. Arredondo F. Echeverry C. Lafon L. (2003). Cell culture protection and *in vivo* neuroprotective capacity of flavonoids. Neurotox. Res. 5, 425–432. 10.1007/BF03033172 14715446

[B57] Darvesh A. S. Carroll R. T. Bishayee A. Novotny N. A. Geldenhuys W. J. VAN DER Schyf C. J. (2012). Curcumin and neurodegenerative diseases: A perspective. Expert Opin. Investig. Drugs 21, 1123–1140. 10.1517/13543784.2012.693479 22668065

[B58] De deurwaerdère P. (2016). Cariprazine: New dopamine biased agonist for neuropsychiatric disorders. Drugs Today 52, 97–110. 10.1358/dot.2016.52.2.2461868 27092339

[B59] De sousa D. P. Quintans L. JR DE Almeida R. N. (2007). Evolution of the anticonvulsant activity of α-terpineol. Pharm. Biol. 45, 69–70. 10.1080/13880200601028388

[B60] Deb S. Phukan B. C. Dutta A. Paul R. Bhattacharya P. Manivasagam T. (2020). Natural products and their therapeutic effect on autism spectrum disorder. Personalized Food Intervention and Therapy for Autism Spectrum Disorder Management, 601–614. 10.1007/978-3-030-30402-7_22 32006376

[B61] Derubeis R. J. Hollon S. D. Amsterdam J. D. Shelton R. C. Young P. R. Salomon R. M. (2005). Cognitive therapy vs medications in the treatment of moderate to severe depression. Arch. Gen. Psychiatry 62, 409–416. 10.1001/archpsyc.62.4.409 15809408

[B62] Di liberto V. Mäkelä J. Korhonen L. Olivieri M. Tselykh T. Mälkiä A. (2012). Involvement of estrogen receptors in the resveratrol-mediated increase in dopamine transporter in human dopaminergic neurons and in striatum of female mice. Neuropharmacology 62, 1011–1018. 10.1016/j.neuropharm.2011.10.010 22041555

[B63] Dickerson F. B. Lehman A. F. (2011). Evidence-based psychotherapy for schizophrenia: 2011 update. J. Nerv. Ment. Dis. 199, 520–526. 10.1097/NMD.0b013e318225ee78 21814072

[B64] Dimpfel W. (2006). Different anticonvulsive effects of hesperidin and its aglycone hesperetin on electrical activity in the rat hippocampus *in-vitro* . J. Pharm. Pharmacol. 58, 375–379. 10.1211/jpp.58.3.0012 16536905

[B65] Dipiro J. T. Talbert R. L. Yee G. C. Matzke G. R. Wells B. G. Posey L. M. (2014). Pharmacotherapy: A pathophysiologic approach. New York: McGraw-Hill Medical.

[B66] Dubowitz H. Prescott L. Feigelman S. Lane W. Kim J. (2008). Screening for intimate partner violence in a pediatric primary care clinic. Pediatrics 121, e85–e91. 10.1542/peds.2007-0904 18166548

[B67] Edition F. (2013). Diagnostic and statistical manual of mental disorders, 21. American Psychiatric Association Publishing.

[B68] Enatescu V. R. Kalinovic R. Vlad G. Nussbaum L. A. Hogea L. Enatescu I. (2020). The presence of peripheral inflammatory markers in patients with major depressive disorder, the associated symptoms profiles and the antidepressant efficacy of celecoxib. Farmacia 68, 483–491. 10.31925/farmacia.2020.3.14

[B69] Evans S. W. Owens J. S. Wymbs B. T. Ray A. R. (2018). Evidence-based psychosocial treatments for children and adolescents with attention deficit/hyperactivity disorder. J. Clin. Child. Adolesc. Psychol. 47, 157–198. 10.1080/15374416.2017.1390757 29257898

[B312] Fang K. Li H. R. Chen X. X. Gao X. R. Huang L. L. Du A. Q. Jiang C. Li H. Ge J. F. (2020). Quercetin alleviates LPS-induced depression-like behavior in rats via regulating BDNF-related imbalance of Copine 6 and TREM1/2 in the hippocampus and PFC. Front. Pharmacol. 10, 1544. 10.3389/fphar.2019.01544 32009956 PMC6978986

[B70] Fava M. Alpert J. E. Carmin C. N. Wisniewski S. R. Trivedi M. H. Biggs M. M. (2004). Clinical correlates and symptom patterns of anxious depression among patients with major depressive disorder in STAR* D. Psychol. Med. 34, 1299–1308. 10.1017/s0033291704002612 15697056

[B71] Felipe F. C. B. Sousa Filho J. T. DE Oliveira Souza L. E. Silveira J. A. DE Andrade Uchoa D. E. Silveira E. R. (2007). Piplartine, an amide alkaloid from Piper tuberculatum, presents anxiolytic and antidepressant effects in mice. Phytomedicine. 14, 605–612. 10.1016/j.phymed.2006.12.015 17399971

[B72] Fernández S. P. Wasowski C. Paladini A. C. Marder M. (2005). Synergistic interaction between hesperidin, a natural flavonoid, and diazepam. Eur. J. Pharmacol. 512, 189–198. 10.1016/j.ejphar.2005.02.039 15840404

[B73] Ferrari A. J. Baxter A. J. Whiteford H. A. (2011). A systematic review of the global distribution and availability of prevalence data for bipolar disorder. J. Affect. Disord. 134, 1–13. 10.1016/j.jad.2010.11.007 21131055

[B74] Fiest K. M. Sauro K. M. Wiebe S. Patten S. B. Kwon C.-S. Dykeman J. (2017). Prevalence and incidence of epilepsy: A systematic review and meta-analysis of international studies. Neurology 88, 296–303. 10.1212/WNL.0000000000003509 27986877 PMC5272794

[B75] Fombonne E. (2009). Epidemiology of pervasive developmental disorders. Pediatr. Res. 65, 591–598. 10.1203/PDR.0b013e31819e7203 19218885

[B76] Fombonne E. (2006). Past and future perspectives on autism epidemiology. 10.1038/sj.mp.4001164 12142933

[B77] Freedman R. (2010). The American psychiatric publishing textbook of psychopharmacology. American Psychiatric Association Publishing.

[B78] Fu M. Sun Z.-H. Zuo H.-C. (2010). Neuroprotective effect of piperine on primarily cultured hippocampal neurons. Biol. Pharm. Bull. 33, 598–603. 10.1248/bpb.33.598 20410592

[B79] Fujimaki K. Morinobu S. Duman R. S. (2000). Administration of a cAMP phosphodiesterase 4 inhibitor enhances antidepressant-induction of BDNF mRNA in rat hippocampus. Neuropsychopharmacology 22, 42–51. 10.1016/S0893-133X(99)00084-6 10633490

[B313] Galeotti N. (2017). *Hypericum perforatum* (St John’s wort) beyond depression: A therapeutic perspective for pain conditions. J. Ethnopharmacol. 200, 136–146. 10.1016/j.jep.2017.02.016 28216196

[B330] Gawel K. Kukula-Koch W. Nieoczym D. Stepnik K. Ent V. W. Banono N. S. (2020). The influence of palmatine isolated from *Berberis sibirica* Radix on pentylenetetrazole-induced seizures in zebrafish. Cells 9 (5), 1233. 10.3390/cells9051233 PMC7290958 32429356

[B328] Gawel K. Kukula-Koch W. Banono N. S. Nieoczym D. Targowska-Duda K. M. Czernicka L. Parada-Turska J. Esguerra C. V. (2021). 6-Gingerol, a major constituent of *Zingiber officinale* rhizoma, exerts anticonvulsant activity in the pentylenetetrazole-induced seizure model in larval zebrafish. Int. J. Mol. Sci. 22 (14), 7745. 10.3390/ijms22147745 34299361 PMC8305044

[B80] Gericke N. P. Van wyk B.-E. (2001b). Pharmaceutical compositions containing mesembrine and related compounds. Google Patents.

[B81] Gericke N. Van wyk B. (2001a). African Natural Health CC. Pharmaceutical compositions containing mesembrine and related compounds.

[B82] Girish C. Raj V. Arya J. Balakrishnan S. (2012). Evidence for the involvement of the monoaminergic system, but not the opioid system in the antidepressant-like activity of ellagic acid in mice. Eur. J. Pharmacol. 682, 118–125. 10.1016/j.ejphar.2012.02.034 22387858

[B83] Goldberg D. Pilling S. Kendall T. Ferrier N. Foster T. Gates J. (2005). Management of depression in primary and secondary care. London, England: Gaskell.

[B84] Golechha M. Chaudhry U. Bhatia J. Saluja D. Arya D. S. (2011). Naringin protects against kainic acid-induced status epilepticus in rats: Evidence for an antioxidant, anti-inflammatory and neuroprotective intervention. Biol. Pharm. Bull. 34, 360–365. 10.1248/bpb.34.360 21372385

[B85] Golechha M. Sarangal V. Bhatia J. Chaudhry U. Saluja D. Arya D. S. (2014). Naringin ameliorates pentylenetetrazol-induced seizures and associated oxidative stress, inflammation, and cognitive impairment in rats: Possible mechanisms of neuroprotection. Epilepsy Behav. 41, 98–102. 10.1016/j.yebeh.2014.09.058 25461197

[B86] Gopi S. Jacob J. Varma K. Jude S. Amalraj A. Arundhathy C. (2017). Comparative oral absorption of curcumin in a natural turmeric matrix with two other curcumin formulations: An open‐label parallel‐arm study. Phytother. Res. 31, 1883–1891. 10.1002/ptr.5931 29027274

[B87] Guenne S. Balmus I. Hilou A. Ouattara N. Kiendrebéogo M. Ciobica A. (2016). The relevance of Asteraceae family plants in most of the neuropsychiatric disorders treatment. Int. J. Phyt 8, 176–182.

[B88] Gullotta F. Schindler F. Schmutzler R. Weeks-Seifert A. (1985). GFAP in brain tumor diagnosis: Possibilities and limitations. Pathol. Res. Pract. 180, 54–60. 10.1016/S0344-0338(85)80075-3 4034433

[B89] Gupta S. Black D. W. Smith D. A. (1994). Risperidone: Review of its pharmacology and therapeutic use in schizophrenia. Ann. Clin. Psychiatry. 6, 173–180. 10.3109/10401239409149000 7533585

[B90] Gutmann H. Bruggisser R. Schaffner W. Bogman K. Botomino A. Drewe J. (2002). Transport of amentoflavone across the blood-brain barrier *in vitro* . Planta Med. 68, 804–807. 10.1055/s-2002-34401 12357391

[B91] Haleagrahara N. Radhakrishnan A. Lee N. Kumar P. (2009). Flavonoid quercetin protects against swimming stress-induced changes in oxidative biomarkers in the hypothalamus of rats. Eur. J. Pharmacol. 621, 46–52. 10.1016/j.ejphar.2009.08.030 19744476

[B92] Han X. H. Hong S. S. Hwang J. S. Lee M. K. Hwang B. Y. Ro J. S. (2007). Monoamine oxidase inhibitory components from Cayratia japonica. Arch. Pharm. Res. 30, 13–17. 10.1007/BF02977772 17328236

[B93] Hanks G. R. (2002). Narcissus and daffodil: The genus Narcissus. CRC Press.

[B94] Hasanzadeh E. Mohammadi M.-R. Ghanizadeh A. Rezazadeh S.-A. Tabrizi M. Rezaei F. (2012). A double-blind placebo controlled trial of Ginkgo biloba added to risperidone in patients with autistic disorders. Child. Psychiatry Hum. Dev. 43, 674–682. 10.1007/s10578-012-0292-3 22392415

[B95] Health N. C. C. F. M. (2009). Attention deficit hyperactivity disorder: Diagnosis and management of ADHD in children, young people and adults. 22420012

[B96] Heinrich M. Appendino G. Efferth T. Fürst R. Izzo A. A. Kayser O. (2020). Best practice in research – overcoming common challenges in phytopharmacological research. J. Ethnopharmacol. 246, 112230. 10.1016/j.jep.2019.112230 31526860

[B97] Heinrich M. Williamson E. M. Gibbons S. Barnes J. Prieto-Garcia J. (2017). Fundamentals of pharmacognosy and phytotherapy E-BOOK. Elsevier Health Sciences.

[B98] Hope J. Castle D. Keks N. A. (2018). Brexpiprazole: A new leaf on the partial dopamine agonist branch. Australas. Psychiatry. 26, 92–94. 10.1177/1039856217732473 29017334

[B99] Hossain R. Quispe C. Herrera-Bravo J. Beltrán J. F. Islam M. T. Shaheen S. (2022). Neurobiological promises of the bitter diterpene lactone andrographolide. Oxid. Med. Cell. Longev. 2022, 3079577. 10.1155/2022/3079577 35154564 PMC8825670

[B100] Hossain R. Sarkar C. Hassan S. M. H. Khan R. A. Arman M. Ray P. (2021). *In silico* screening of natural products as potential inhibitors of SARS-CoV-2 using molecular docking simulation. Chin. J. Integr. Med. 28, 249–256. 10.1007/s11655-021-3504-5 34913151 PMC8672856

[B101] Hosseinzadeh H. Noraei N. B. (2009). Anxiolytic and hypnotic effect of Crocus sativus aqueous extract and its constituents, crocin and safranal, in mice. Phytother. Res. 23, 768–774. 10.1002/ptr.2597 19142981

[B102] Houslay M. D. Schafer P. Zhang K. Y. (2005). Keynote review: phosphodiesterase-4 as a therapeutic target. Drug Discov. Today 10, 1503–1519. 10.1016/S1359-6446(05)03622-6 16257373

[B327] Hsu C. D. Hsieh L. H. Chen Y. L. Lin I. C. Chen Y. R. Chen C. C. Shirakawa H. Yang S. C. (2021). Complementary effects of pine bark extract supplementation on inattention, impulsivity, and antioxidative status in children with attention-deficit hyperactivity disorder: A double-blinded randomized placebo-controlled cross-over study. Phytother. Res. 35 (6), 3226–3235. 10.1002/ptr.7036 33559134

[B103] Huang C.-W. Chow J. C. Tsai J.-J. Wu S.-N. (2012). Characterizing the effects of Eugenol on neuronal ionic currents and hyperexcitability. Psychopharmacology 221, 575–587. 10.1007/s00213-011-2603-y 22160139

[B104] Huen M. S. Leung J. W. Ng W. Lui W. Chan M. N. Wong J. T.-F. (2003). 5, 7-Dihydroxy-6-methoxyflavone, a benzodiazepine site ligand isolated from Scutellaria baicalensis Georgi, with selective antagonistic properties. Biochem. Pharmacol. 66, 125–132. 10.1016/s0006-2952(03)00233-8 12818372

[B105] Ishola I. O. Chatterjee M. Tota S. Tadigopulla N. Adeyemi O. O. Palit G. (2012). Antidepressant and anxiolytic effects of amentoflavone isolated from *Cnestis ferruginea* in mice. Pharmacol. Biochem. Behav. 103, 322–331. 10.1016/j.pbb.2012.08.017 22944105

[B106] Israelyan N. Margolis K. G. (2019). Reprint of: Serotonin as a link between the gut-brain-microbiome axis in autism spectrum disorders. Pharmacol. Res. 140, 115–120. 10.1016/j.phrs.2018.12.023 30658882 PMC6366671

[B107] Jain A. (2020). Review article- bipolar disorder: Diagnosis, pathophysiology and therapy.

[B108] Jann M. W. (2014). Diagnosis and treatment of bipolar disorders in adults: A review of the evidence on pharmacologic treatments. Am. Health Drug Benefits 7, 489–499. 25610528 PMC4296286

[B109] Jeong K. H. Jung U. J. Kim S. R. (2015). Naringin attenuates autophagic stress and neuroinflammation in kainic acid-treated hippocampus *in vivo* . Evidence-Based Complementary Altern. Med., 1–9. 10.1155/2015/354326 PMC4466392 26124853

[B110] Ji N. Y. Findling R. L. (2015). An update on pharmacotherapy for autism spectrum disorder in children and adolescents. Curr. Opin. Psychiatry 28, 91–101. 10.1097/YCO.0000000000000132 25602248

[B111] Jiang Z. Guo M. Shi C. Wang H. Yao L. Liu L. (2015). Protection against cognitive impairment and modification of epileptogenesis with curcumin in a post-status epilepticus model of temporal lobe epilepsy. Neuroscience 310, 362–371. 10.1016/j.neuroscience.2015.09.058 26415768

[B112] Johnson C. P. Myers S. M. American Academy of Pediatrics Council on Children With Disabilities (2007). Identification and evaluation of children with autism spectrum disorders. Pediatrics 120, 1183–1215. 10.1542/peds.2007-2361 17967920

[B113] Kanner A. M. Bicchi M. M. (2022). Antiseizure medications for adults with epilepsy: A review. Jama 327, 1269–1281. 10.1001/jama.2022.3880 35380580

[B114] Kaur R. Chopra K. Singh D. (2007). Role of alpha2 receptors in quercetin-induced behavioral despair in mice. J. Med. Food 10, 165–168. 10.1089/jmf.2005.063 17472482

[B115] Kavvadias D. Sand P. Youdim K. A. Qaiser M. Z. Rice‐Evans C. Baur R. (2004). The flavone hispidulin, a benzodiazepine receptor ligand with positive allosteric properties, traverses the blood–brain barrier and exhibits anticonvulsive effects. Br. J. Pharmacol. 142, 811–820. 10.1038/sj.bjp.0705828 15231642 PMC1575061

[B116] Kawabata K. Kawai Y. Terao J. (2010). Suppressive effect of quercetin on acute stress-induced hypothalamic-pituitary-adrenal axis response in Wistar rats. J. Nutr. Biochem. 21, 374–380. 10.1016/j.jnutbio.2009.01.008 19423323

[B117] Kazmi I. Gupta G. Afzal M. Anwar F. (2012). Anticonvulsant and depressant-like activity of ursolic acid stearoyl glucoside isolated from Lantana camara L.(verbanaceae). Asian Pac. J. Trop. Dis. 2, S453–S456. 10.1016/s2222-1808(12)60202-3

[B118] Kelvin E. A. Hesdorffer D. C. Bagiella E. Andrews H. Pedley T. A. Shih T. T. (2007). Prevalence of self-reported epilepsy in a multiracial and multiethnic community in New York City. Epilepsy Res. 77, 141–150. 10.1016/j.eplepsyres.2007.09.012 18023147

[B119] Kessler R. C. Chiu W. T. Demler O. Walters E. E. (2005). Prevalence, severity, and comorbidity of 12-month DSM-IV disorders in the national comorbidity survey replication. Arch. Gen. Psychiatry 62, 617–627. 10.1001/archpsyc.62.6.617 15939839 PMC2847357

[B120] Kilpatrick J. Swafford J. Findell B. Council N. R. (2001). Adding it up: Helping children learn mathematics. Citeseer.

[B121] Komossa K. Depping A. M. Gaudchau A. Kissling W. Leucht S. (2010). Second‐generation antipsychotics for major depressive disorder and dysthymia. Cochrane Database Syst. Rev. 10.1002/14651858.cd008121.pub2 PMC11994262 21154393

[B122] Kritas S. Saggini A. Varvara G. Murmura G. Caraffa A. Antinolfi P. (2013). Luteolin inhibits mast cell-mediated allergic inflammation. J. Biol. Regul. Homeost. Agents 27, 955–959. 24382176

[B123] Kuhn B. N. Kalivas P. W. Bobadilla A.-C. (2019). Understanding addiction using animal models. Front. Behav. Neurosci. 13, 262. 10.3389/fnbeh.2019.00262 31849622 PMC6895146

[B124] Kulkarni S. K. Bhutani M. K. Bishnoi M. (2008). Antidepressant activity of curcumin: Involvement of serotonin and dopamine system. Psychopharmacology 201, 435–442. 10.1007/s00213-008-1300-y 18766332

[B125] Kumar A. Lalitha S. Mishra J. (2014). Hesperidin potentiates the neuroprotective effects of diazepam and gabapentin against pentylenetetrazole-induced convulsions in mice: Possible behavioral, biochemical and mitochondrial alterations. Indian J. Pharmacol. 46, 309–315. 10.4103/0253-7613.132180 24987179 PMC4071709

[B126] Kumar A. Lalitha S. Mishra J. (2013). Possible nitric oxide mechanism in the protective effect of hesperidin against pentylenetetrazole (PTZ)-induced kindling and associated cognitive dysfunction in mice. Epilepsy Behav. 29, 103–111. 10.1016/j.yebeh.2013.06.007 23939034

[B127] Kumaravel P. Melchias G. Vasanth N. Manivasagam T. (2017). Epigallocatechin gallate attenuates behavioral defects in sodium valproate induced autism rat model. Res. J. Pharm. Technol. 10, 1477–1480. 10.5958/0974-360x.2017.00260.8

[B128] Kunnumakkara A. B. Bordoloi D. Padmavathi G. Monisha J. Roy N. K. Prasad S. (2017). Curcumin, the golden nutraceutical: Multitargeting for multiple chronic diseases. Br. J. Pharmacol. 174, 1325–1348. 10.1111/bph.13621 27638428 PMC5429333

[B129] Kwon C. S. Wagner R. G. Carpio A. Jetté N. Newton C. R. Thurman D. J. (2022). The worldwide epilepsy treatment gap: A systematic review and recommendations for revised definitions - a report from the ilae epidemiology commission. Epilepsia 63, 551–564. 10.1111/epi.17112 35001365

[B130] Kwon S.-H. Lee H.-K. Kim J.-A. Hong S.-I. Kim H.-C. Jo T.-H. (2010). Neuroprotective effects of chlorogenic acid on scopolamine-induced amnesia via anti-acetylcholinesterase and anti-oxidative activities in mice. Eur. J. Pharmacol. 649, 210–217. 10.1016/j.ejphar.2010.09.001 20854806

[B131] Lake J. (2000). Natural product-derived treatments of neuropsychiatric disorders: Review of progress and recommendations. Stud. Nat. Prod. Chem. 24, 1093–1137.

[B132] Lam T. K. Shao S. Zhao Y. Marincola F. Pesatori A. Bertazzi P. A. (2012). Influence of quercetin-rich food intake on microRNA expression in lung cancer tissues. Cancer Epidemiol. Biomarkers Prev. 21, 2176–2184. 10.1158/1055-9965.EPI-12-0745 23035181 PMC3538163

[B133] Landa R. J. (2008). Diagnosis of autism spectrum disorders in the first 3 years of life. Nat. Clin. Pract. Neurol. 4, 138–147. 10.1038/ncpneuro0731 18253102

[B134] Lane R. Baldwin D. (1997). Selective serotonin reuptake inhibitor-induced serotonin syndrome: Review. J. Clin. Psychopharmacol. 17, 208–221. 10.1097/00004714-199706000-00012 9169967

[B135] Langguth B. Bär R. Wodarz N. Wittmann M. Laufkötter R. (2011). Correspondence (letter to the editor): Paradoxical reaction in ADHD. Dtsch. Arztebl. Int. 108, 541. 10.3238/arztebl.2011.0541a 21886668 PMC3163785

[B136] Lavoie F. W. Gansert G. G. Weiss R. E. (1990). Value of initial ECG findings and plasma drug levels in cyclic antidepressant overdose. Ann. Emerg. Med. 19, 696–700. 10.1016/s0196-0644(05)82482-5 2188541

[B137] Lavretsky H. (2008). History of schizophrenia as a psychiatric disorder. Clin. Handb. schizophrenia 1.

[B138] Leskovec T. J. Rowles B. M. Findling R. L. (2008). Pharmacological treatment options for autism spectrum disorders in children and adolescents. Harv. Rev. Psychiatry 16, 97–112. 10.1080/10673220802075852 18415882

[B139] Levy S. E. Ds M. (2009). Autism. Lancet 374, 1627–1638. 10.1016/S0140-6736(09)61376-3 19819542 PMC2863325

[B140] Li Y.-C. Wang F.-M. Pan Y. Qiang L.-Q. Cheng G. Zhang W.-Y. (2009). Antidepressant-like effects of curcumin on serotonergic receptor-coupled AC-cAMP pathway in chronic unpredictable mild stress of rats. Prog. Neuropsychopharmacol. Biol. Psychiatry 33, 435–449. 10.1016/j.pnpbp.2009.01.006 19302828

[B315] Li R. Wang X. Qin T. Qu R. Ma S. (2016). Apigenin ameliorates chronic mild stress-induced depressive behavior by inhibiting interleukin-1β production and NLRP3 inflammasome activation in the rat brain. Behav. Brain Res. 296, 318–325. 10.1016/j.bbr.2015.09.031 26416673

[B141] Lim D. W. Han T. Jung J. Song Y. Um M. Y. Yoon M. (2018). Chlorogenic Acid from Hawthorn berry (*Crataegus pinnatifida* fruit) prevents stress hormone‐induced depressive behavior, through monoamine oxidase b‐reactive oxygen species signaling in hippocampal astrocytes of mice. Mol. Nutr. Food Res. 62, 1800029. 10.1002/mnfr.201800029 29893510

[B142] Lin M.-T. Wang J.-J. Young M.-S. (2002). The protective effect of dl-tetrahydropalmatine against the development of amygdala kindling seizures in rats. Neurosci. Lett. 320, 113–116. 10.1016/s0304-3940(01)02508-3 11852175

[B143] Lin T.-Y. Lu C.-W. Wang C.-C. Lu J.-F. Wang S.-J. (2012). Hispidulin inhibits the release of glutamate in rat cerebrocortical nerve terminals. Toxicol. Appl. Pharmacol. 263, 233–243. 10.1016/j.taap.2012.06.015 22759588

[B144] Lin T. Y. Lu C. W. Wang C.-C. Wang Y.-C. Wang S.-J. (2011). Curcumin inhibits glutamate release in nerve terminals from rat prefrontal cortex: Possible relevance to its antidepressant mechanism. Prog. Neuropsychopharmacol. Biol. Psychiatry 35, 1785–1793. 10.1016/j.pnpbp.2011.06.012 21741425

[B145] Liu Y.-F. Gao F. Li X.-W. Jia R.-H. Meng X.-D. Zhao R. (2012). The anticonvulsant and neuroprotective effects of baicalin on pilocarpine-induced epileptic model in rats. Neurochem. Res. 37, 1670–1680. 10.1007/s11064-012-0771-8 22528832

[B146] London E. (2007). The role of the neurobiologist in redefining the diagnosis of autism. Brain Pathol. 17, 408–411. 10.1111/j.1750-3639.2007.00103.x 17919126 PMC8095627

[B147] Lopez P. L. Torrente F. M. Ciapponi A. Lischinsky A. G. Cetkovich‐Bakmas M. Rojas J. I. (2018). Cognitive‐behavioural interventions for attention deficit hyperactivity disorder (ADHD) in adults. Cochrane Database Syst. Rev. 10.1002/14651858.cd010840.pub2 PMC6494390 29566425

[B148] Luduvico K. P. Spohr L. Soares M. S. P. Teixeira F. C. DE Farias A. S. Bona N. P. (2020). Antidepressant effect and modulation of the redox system mediated by tannic acid on lipopolysaccharide-induced depressive and inflammatory changes in mice. Neurochem. Res. 45, 2032–2043. 10.1007/s11064-020-03064-5 32500408

[B329] Kanazawa L. K. S. Débora D. V. Etiéli W. M. Hocayen P. de A. S. dos Reis Lívero F. A. Stipp M. C. (2016). Quercetin reduces manic-like behavior and brain oxidative stress induced by paradoxical sleep deprivation in mice. Free Rad. Biol. Med. 99, 79–86. 10.1016/j.freeradbiomed.2016.07.027 27475725

[B325] Kanazawa L. K. Vecchia D. D. Wendler E. M. Hocayen P. A. Beirão P. S. de Mélo M. L. (2017). Effects of acute and chronic quercetin administration on methylphenidate-induced hyperlocomotion and oxidative stress. Life Sci. 171, 1–8. 10.1016/j.lfs.2017.01.007 28104366

[B326] Kean J. D. Downey L. A. Sarris J. Kaufman J. Zangara A. Stough C. (2022). Effects of Bacopa monnieri (CDRI 08®) in a population of males exhibiting inattention and hyperactivity aged 6 to 14 years: A randomized, double-blind, placebo-controlled trial. Phytother. Res. 36 (2), 996–1012. 10.1002/ptr.7372 35041248

[B149] Lüllmann H. Mohr K. (2006). Pharmakologie und Toxikologie: Arzneimittelwirkungen verstehen-Medikamente gezielt einsetzen; ein Lehrbuch für Studierende der Medizin, der Pharmazie und der Biowissenschaften, eine Informationsquelle für Ärzte, Apotheker und Gesundheitspolitiker; 129 Tabellen. Georg Thieme Verlag.

[B150] Luszczki J. J. Andres-Mach M. Cisowski W. Mazol I. Glowniak K. Czuczwar S. J. (2009). Osthole suppresses seizures in the mouse maximal electroshock seizure model. Eur. J. Pharmacol. 607, 107–109. 10.1016/j.ejphar.2009.02.022 19236860

[B151] Łuszczki J. J. Andres-Mach M. Gleńsk M. Skalicka-Woźniak K. (2010). Anticonvulsant effects of four linear furanocoumarins, bergapten, imperatorin, oxypeucedanin, and xanthotoxin, in the mouse maximal electroshock-induced seizure model: A comparative study. Pharmacol. Rep. 62, 1231–1236. 10.1016/s1734-1140(10)70387-x 21273683

[B152] Lyon M. R. Cline J. C. DE Zepetnek J. T. Shan J. J. Pang P. Benishin C. (2001). Effect of the herbal extract combination panax quinquefolium and *Ginkgo biloba* on attention-deficit hyperactivity disorder: A pilot study. J. Psychiatry Neurosci. 26, 221–228. 11394191 PMC1408291

[B153] Machado D. G. Bettio L. E. Cunha M. P. Santos A. R. Pizzolatti M. G. Brighente I. M. (2008). Antidepressant-like effect of rutin isolated from the ethanolic extract from *Schinus molle* L. In mice: Evidence for the involvement of the serotonergic and noradrenergic systems. Eur. J. Pharmacol. 587, 163–168. 10.1016/j.ejphar.2008.03.021 18457827

[B154] Maiga A. Diallo D. Fane S. Sanogo R. Paulsen B. S. Cisse B. (2005). A survey of toxic plants on the market in the district of bamako, Mali: Traditional knowledge compared with a literature search of modern pharmacology and toxicology. J. Ethnopharmacol. 96, 183–193. 10.1016/j.jep.2004.09.005 15588669

[B320] Mitra S. Anjum J. Muni M. Das R. Rauf A. Islam F. (2022). Exploring the journey of emodin as a potential neuroprotective agent: Novel therapeutic insights with molecular mechanism of action. Biomed. Pharmacother. 149, 112877. 10.1016/j.biopha.2022.112877 35367766

[B155] Malhi G. Adams D. Lampe L. Paton M. O’Connor N. Newton L. (2009). Clinical practice recommendations for bipolar disorder. Acta Psychiatr. Scand. 119, 27–46. 10.1111/j.1600-0447.2009.01383.x 19356155

[B156] Mandah S. N. Osuagwu C. E. (2020). Characteristics behaviours and factors responsible for attention deficit hyperactivity disorder (ADHD) among senior secondary school students in rivers state, Nigeria. Eur. J. Special Educ. Res. 6.

[B157] Manji H. K. Drevets W. C. Charney D. S. (2001). The cellular neurobiology of depression. Nat. Med. 7, 541–547. 10.1038/87865 11329053

[B158] Mann J. J. Currier D. (2006). Effects of genes and stress on the neurobiology of depression. Int. Rev. Neurobiol. 73, 153–189. 10.1016/S0074-7742(06)73005-7 16737904

[B159] Marder S. R. Meibach R. C. (1994). Risperidone in the treatment of schizophrenia. Am. J. Psychiatry 151, 825–835. 10.1176/ajp.151.6.825 7514366

[B160] Martens G. Van loo K. (2007). Genetic and environmental factors in complex neurodevelopmental disorders. Curr. Genomics 8, 429–444. 10.2174/138920207783591717 19412416 PMC2647153

[B161] Martin C. A. Nuzzo P. A. Ranseen J. D. Kleven M. S. Guenthner G. Williams Y. (2018). Lobeline effects on cognitive performance in adult ADHD. J. Atten. Disord. 22, 1361–1366. 10.1177/1087054713497791 23966351 PMC4062608

[B162] Martin-mcgill K. J. Bresnahan R. Levy R. G. Cooper P. N. (2020). Ketogenic diets for drug‐resistant epilepsy. Cochrane Database Syst. Rev. 10.1002/14651858.cd001903.pub5 PMC7387249 32588435

[B163] Mcguffin P. Rijsdijk F. Andrew M. Sham P. Katz R. Cardno A. (2003). The heritability of bipolar affective disorder and the genetic relationship to unipolar depression. Arch. Gen. Psychiatry 60, 497–502. 10.1001/archpsyc.60.5.497 12742871

[B164] Medina J. H. Paladini A. C. Wolfman C. DE Stein M. L. Calvo D. Diaz L. E. (1990). Chrysin (5, 7-di-OH-flavone), a naturally-occurring ligand for benzodiazepine receptors, with anticonvulsant properties. Biochem. Pharmacol. 40, 2227–2231. 10.1016/0006-2952(90)90716-x 2173925

[B165] Menza M. Dobkin R. D. Marin H. Mark M. Gara M. Buyske S. (2009). A controlled trial of antidepressants in patients with Parkinson disease and depression. Neurology 72, 886–892. 10.1212/01.wnl.0000336340.89821.b3 19092112 PMC2677475

[B322] Miodownik C. Lerner V. Kudkaeva N. Lerner P. P. Pashinian A. Bersudsky Y. (2019). Curcumin as add-on to antipsychotic treatment in patients with chronic schizophrenia: A randomized, double-blind, placebo-controlled study. Clin. Neuropharmacol. 42 (4), 117–122. 10.1097/WNF.0000000000000344 31045590

[B166] Miura T. Noma H. Furukawa T. A. Mitsuyasu H. Tanaka S. Stockton S. (2014). Comparative efficacy and tolerability of pharmacological treatments in the maintenance treatment of bipolar disorder: A systematic review and network meta-analysis. Lancet. Psychiatry 1, 351–359. 10.1016/S2215-0366(14)70314-1 26360999

[B167] Mo J. Guo Y. Yang Y.-S. Shen J.-S. Jin G.-Z. Zhen X. (2007). Recent developments in studies of l-stepholidine and its analogs: Chemistry, pharmacology and clinical implications. Curr. Med. Chem. 14, 2996–3002. 10.2174/092986707782794050 18220736

[B168] Mohr P. Pecenak J. Svestka J. Swingler D. Treuer T. (2005). Treatment of acute agitation in psychotic disorders. Neuro Endocrinol. Lett. 26, 327–335. 16136016

[B169] Möller H.-J. (2005). Risperidone: A review. Expert Opin. Pharmacother. 6, 803–818. 10.1517/14656566.6.5.803 15934906

[B170] Mondiale de la santé A. (2013). Projet de plan d’action pour la lutte contre les maladies non transmissibles 2013-2020: Rapport du Secrétariat.

[B171] Moore A. R. O’keeffe S. T. (1999). Drug-induced cognitive impairment in the elderly. Drugs Aging 15, 15–28. 10.2165/00002512-199915010-00002 10459729

[B172] Moreira A. L. R. VAN Meter A. Genzlinger J. Youngstrom E. A. (2017). Review and meta-analysis of epidemiologic studies of adult bipolar disorder. J. Clin. Psychiatry 78, e1259–e1269. 10.4088/JCP.16r11165 29188905

[B173] Murray R. M. Sham P. VAN Os J. Zanelli J. Cannon M. Mcdonald C. (2004). A developmental model for similarities and dissimilarities between schizophrenia and bipolar disorder. Schizophr. Res. 71, 405–416. 10.1016/j.schres.2004.03.002 15474912

[B174] Nakazawa T. Yasuda T. Ueda J. Ohsawa K. (2003). Antidepressant-like effects of apigenin and 2, 4, 5-trimethoxycinnamic acid from Perilla frutescens in the forced swimming test. Biol. Pharm. Bull. 26, 474–480. 10.1248/bpb.26.474 12673028

[B175] Napoletano M. Norcini G. Pellacini F. Marchini F. Morazzoni G. Ferlenga P. (2001). Phthalazine PDE4 inhibitors. Part 2: The synthesis and biological evaluation of 6-methoxy-1, 4-disubstituted derivatives. Bioorg. Med. Chem. Lett. 11, 33–37. 10.1016/s0960-894x(00)00587-4 11140727

[B176] Nassiri-asl M. Shariati-Rad S. Zamansoltani F. (2008). Anticonvulsive effects of intracerebroventricular administration of rutin in rats. Prog. Neuropsychopharmacol. Biol. Psychiatry 32, 989–993. 10.1016/j.pnpbp.2008.01.011 18262708

[B177] Nestler E. J. Barrot M. Dileone R. J. Eisch A. J. Gold S. J. Monteggia L. M. (2002). Neurobiology of depression. Neuron 34, 13–25. 10.1016/s0896-6273(02)00653-0 11931738

[B178] Nevitt S. J. Marson A. G. Smith C. T. Tudur Smith C. (2019). Carbamazepine versus phenytoin monotherapy for epilepsy: An individual participant data review. Cochrane Database Syst. Rev. 10.1002/14651858.cd001911.pub3 PMC6637502 31318037

[B179] Nevitt S. J. Marson A. G. Weston J. Smith C. T. (2018). Sodium valproate versus phenytoin monotherapy for epilepsy: An individual participant data review. Cochrane Database Syst. Rev. 10.1002/14651858.cd001769.pub4 PMC6513104 30091458

[B180] Newschaffer C. J. Croen L. A. Daniels J. Giarelli E. Grether J. K. Levy S. E. (2007). The epidemiology of autism spectrum disorders. Annu. Rev. Public Health 28, 235–258. 10.1146/annurev.publhealth.28.021406.144007 17367287

[B181] Newton C. R. Garcia H. H. (2012). Epilepsy in poor regions of the world. Lancet 380, 1193–1201. 10.1016/S0140-6736(12)61381-6 23021288

[B323] Nogoceke F. P. Barcaro I. M. R. de Sousa D. P. Andreatini R. (2016). Antimanic-like effects of (R)-(−)-carvone and (S)-(+)-carvone in mice. Neurosci. Lett. 619, 43–48. 10.1016/j.neulet.2016.03.013 26970377

[B182] Nourbala A. Akhoundzadeh S. 2006. Attention-deficit/hyperactivity disorder: etiology and pharmacotherapy.

[B183] Nussbaum L. Hogea L. M. Calina D. Andreescu N. Gradinaru R. Stefanescu R. (2017). Modern treatment approaches in psychoses. PHARMACOGENETIC, neuroimagistic and clinical implications. Farmacia 65, 75–81.

[B184] Olsen H. T. Stafford G. I. VAN Staden J. Christensen S. B. Jäger A. K. (2008). Isolation of the MAO-inhibitor naringenin from Mentha aquatica L. J. Ethnopharmacol. 117, 500–502. 10.1016/j.jep.2008.02.015 18372132

[B185] World Health Organization (1992). The ICD-10 classification of mental and behavioural disorders: Clinical descriptions and diagnostic guidelines. Available at: https://apps.who.int/iris/handle/10665/37958 .

[B186] Otte C. Gold S. M. Penninx B. W. Pariante C. M. Etkin A. Fava M. (2016). Major depressive disorder. Nat. Rev. Dis. Prim. 2, 16065. 10.1038/nrdp.2016.65 27629598

[B319] Pandy V. Vijeepallam K. (2017). Antipsychotic-like activity of scopoletin and rutin against the positive symptoms of schizophrenia in mouse models. Exp. Anim. 66 (4), 417–423. 10.1538/expanim.17-0050 28701621 PMC5682354

[B187] Painuli S. Quispe C. Herrera-Bravo J. Semwal P. Martorell M. Almarhoon Z. M. (2022). Nutraceutical profiling, bioactive composition, and biological applications of *Lepidium sativum* L. Oxid. Med. Cell. Longev. 2022, 2910411. 10.1155/2022/2910411 35096265 PMC8791756

[B188] Papakostas G. I. (2010). The efficacy, tolerability, and safety of contemporary antidepressants. J. Clin. Psychiatry 71, e03–0. 10.4088/JCP.9058se1c.03gry 20371030

[B189] Park H. G. Yoon S. Y. Choi J. Y. Lee G. S. Choi J. H. Shin C. Y. (2007). Anticonvulsant effect of wogonin isolated from Scutellaria baicalensis. Eur. J. Pharmacol. 574, 112–119. 10.1016/j.ejphar.2007.07.011 17692312

[B190] Park H. R. Kong K. H. Yu B. P. Mattson M. P. Lee J. (2012). Resveratrol inhibits the proliferation of neural progenitor cells and hippocampal neurogenesis. J. Biol. Chem. 287, 42588–42600. 10.1074/jbc.M112.406413 23105098 PMC3522260

[B191] Park S.-H. Sim Y.-B. Han P.-L. Lee J.-K. Suh H.-W. (2010). Antidepressant-like effect of chlorogenic acid isolated from Artemisia capillaris Thunb. Animal cells Syst. 14, 253–259. 10.1080/19768354.2010.528192

[B192] Paul B. D. Snyder S. H. (2019). Therapeutic applications of cysteamine and cystamine in neurodegenerative and neuropsychiatric diseases. Front. Neurol. 10, 1315. 10.3389/fneur.2019.01315 31920936 PMC6920251

[B193] Paul S. M. Extein I. Calil H. M. Potter W. Z. Chodoff P. Goodwin F. K. (1981). Use of ECT with treatment-resistant depressed patients at the national institute of mental health. Am. J. Psychiatry 138, 486–489. 10.1176/ajp.138.4.486 6111228

[B194] Plantlist T. (2021). The plant List. Available: http://www.theplantlist.org/(Accessed, 2021).

[B195] Pragnya B. Kameshwari J. Veeresh B. (2014). Ameliorating effect of piperine on behavioral abnormalities and oxidative markers in sodium valproate induced autism in BALB/C mice. Behav. Brain Res. 270, 86–94. 10.1016/j.bbr.2014.04.045 24803211

[B196] Preskorn S. H. Simpson S. (1982). Tricyclic-antidepressant-induced delirium and plasma drug concentration. Am. J. Psychiatry 139, 822–823. 10.1176/ajp.139.6.822 7081500

[B197] Prudic J. Haskett R. F. Mulsant B. Malone K. M. Pettinati H. M. Stephens S. (1996). Resistance to antidepressant medications and short-term clinical response to ECT. Am. J. Psychiatry 153, 985–992. 10.1176/ajp.153.8.985 8678194

[B198] Pyrzanowska J. Piechal A. Blecharz-Klin K. Joniec-Maciejak I. Zobel A. Widy-Tyszkiewicz E. (2012). Influence of long-term administration of rutin on spatial memory as well as the concentration of brain neurotransmitters in aged rats. Pharmacol. Rep. 64, 808–816. 10.1016/s1734-1140(12)70876-9 23087133

[B199] Qin T. Fang F. Song M. Li R. Ma Z. Ma S. (2017). Umbelliferone reverses depression-like behavior in chronic unpredictable mild stress-induced rats by attenuating neuronal apoptosis via regulating ROCK/Akt pathway. Behav. Brain Res. 317, 147–156. 10.1016/j.bbr.2016.09.039 27646771

[B200] Quetglas-llabrés M. M. Quispe C. Herrera-Bravo J. Catarino M. D. Pereira O. R. Cardoso S. M. (2022). Pharmacological properties of bergapten: Mechanistic and therapeutic aspects. Oxid. Med. Cell. Longev. 2022, 8615242. 10.1155/2022/8615242 35509838 PMC9060977

[B201] Quintans-júnior L. J. Guimarães A. G. Araújo B. E. Oliveira G. F. Santana M. T. Moreira F. V. (2010). Carvacrol, (-)-borneol and citral reduce convulsant activity in rodents. Afr. J. Biotechnol. 9, 6566–6572.

[B202] Quispe C. Herrera-Bravo J. Javed Z. Khan K. Raza S. Gulsunoglu-Konuskan Z. (2022). Therapeutic applications of curcumin in diabetes: A review and perspective. Biomed. Res. Int. 2022, 1375892. 10.1155/2022/1375892 35155670 PMC8828342

[B203] Quitkin F. M. Liebowitz M. R. Stewart J. W. Mcgrath P. J. Harrison W. Rabkin J. G. (1984). l-Deprenyl in atypical depressives. Arch. Gen. Psychiatry 41, 777–781. 10.1001/archpsyc.1984.01790190051006 6430257

[B204] Quitkin F. M. Stewart J. W. Mcgrath P. J. Liebowitz M. R. Harrison W. M. Tricamo E. (1988). Phenelzine versus imipramine in the treatment of probable atypical depression: Defining syndrome boundaries of selective MAOI responders. Am. J. Psychiatry 145, 306–311. 10.1176/ajp.145.3.306 3278631

[B205] Rajib H. Muhammad Torequl I. Pranta R. Divya J. Abu Saim Mohammad S. Lutfun N. (2021). Amentoflavone, new hope against SARS-CoV-2: An outlook through its scientific records and an *in silico* study. Pharmacogn. Res. 13, 149–157. 10.5530/pres.13.3.7

[B206] Ramos-Hryb A. B. Cunha M. P. Kaster M. P. Rodrigues A. L. S. (2018). Natural polyphenols and terpenoids for depression treatment: Current status. Stud. Nat. Prod. Chem. 55, 181–221. 10.1016/b978-0-444-64068-0.00006-1

[B207] Rapin I. Tuchman R. F. (2008). Autism: Definition, neurobiology, screening, diagnosis. Pediatr. Clin. North Am. 55, 1129–1146. 10.1016/j.pcl.2008.07.005 18929056

[B208] Ravindran L. N. Stein M. B. (2010). The pharmacologic treatment of anxiety disorders: A review of progress. J. Clin. Psychiatry 71, 839–854. 10.4088/jcp.10r06218blu 20667290

[B209] Raygude K. S. Kandhare A. D. Ghosh P. Bodhankar S. L. (2012). Anticonvulsant effect of fisetin by modulation of endogenous biomarkers. Biomed. Prev. Nutr. 2, 215–222. 10.1016/j.bionut.2012.04.005

[B210] Raza S. S. Khan M. M. Ahmad A. Ashafaq M. Khuwaja G. Tabassum R. (2011). Hesperidin ameliorates functional and histological outcome and reduces neuroinflammation in experimental stroke. Brain Res. 1420, 93–105. 10.1016/j.brainres.2011.08.047 21959178

[B211] Reaven J. Blakeley‐Smith A. Culhane‐Shelburne K. Hepburn S. (2012). Group cognitive behavior therapy for children with high‐functioning autism spectrum disorders and anxiety: A randomized trial. J. Child. Psychol. Psychiatry 53, 410–419. 10.1111/j.1469-7610.2011.02486.x 22435114 PMC4392045

[B212] Reddy H. M. Poole J. S. Maguire G. A. Stahl S. M. (2020). New medications for neuropsychiatric disorders. Psychiatr. Clin. North Am. 43, 399–413. 10.1016/j.psc.2020.02.008 32439029

[B324] Recart V. M. Spohr L. Soares M. S. P. Mattos B. d. S. Bona N. P. Pedra N. S. (2021). Gallic acid protects cerebral cortex, hippocampus, and striatum against oxidative damage and cholinergic dysfunction in an experimental model of manic-like behavior: Comparison with lithium effects. Int. J. Dev. Neurosci. 81, 167–178. 10.1002/jdn.10086 33394512

[B213] Rossignol D. A. Frye R. E. (2014). Evidence linking oxidative stress, mitochondrial dysfunction, and inflammation in the brain of individuals with autism. Front. Physiol. 5, 150. 10.3389/fphys.2014.00150 24795645 PMC4001006

[B214] Ryvlin P. Cross J. H. Rheims S. (2014). Epilepsy surgery in children and adults. Lancet. Neurol. 13, 1114–1126. 10.1016/S1474-4422(14)70156-5 25316018

[B215] Sakurada T. Kuwahata H. Katsuyama S. Komatsu T. Morrone L. A. Corasaniti M. T. (2009). Intraplantar injection of bergamot essential oil into the mouse hindpaw: Effects on capsaicin‐induced nociceptive behaviors. Int. Rev. Neurobiol. 85, 237–248. 10.1016/S0074-7742(09)85018-6 19607974

[B216] Salehi B. Calina D. Docea A. O. Koirala N. Aryal S. Lombardo D. (2020). Curcumin's nanomedicine formulations for therapeutic application in neurological diseases. J. Clin. Med. 9, E430. 10.3390/jcm9020430 32033365 PMC7074182

[B217] Salehi B. Imani R. Mohammadi M. R. Fallah J. Mohammadi M. Ghanizadeh A. (2010). Ginkgo biloba for attention-deficit/hyperactivity disorder in children and adolescents: A double blind, randomized controlled trial. Prog. Neuropsychopharmacol. Biol. Psychiatry 34, 76–80. 10.1016/j.pnpbp.2009.09.026 19815048

[B218] Salehi B. Jornet P. L. Lopez E. P. F. Calina D. Sharifi-Rad M. Ramirez-Alarcon K. (2019a). Plant-Derived bioactives in oral mucosal lesions: A key emphasis to curcumin, lycopene, chamomile, aloe vera, green tea and coffee properties. Biomolecules 9, E106. 10.3390/biom9030106 30884918 PMC6468600

[B219] Salehi B. Sestito S. Rapposelli S. Peron G. Calina D. Sharifi-Rad M. (2019b). Epibatidine: A promising natural alkaloid in health. Biomolecules 9, 6. 10.3390/biom9010006 PMC6359223 30583611

[B221] Sarris J. Marx W. Ashton M. M. Ng C. H. Galvao-Coelho N. Ayati Z. (2021). Plant-based medicines (phytoceuticals) in the treatment of psychiatric disorders: A meta-review of meta-analyses of randomized controlled trials: Les médicaments à base de plantes (phytoceutiques) dans le traitement des troubles psychiatriques: Une méta-revue des méta-analyses d'essais randomisés contrôlés. Can. J. Psychiatry. 66, 849–862. 10.1177/0706743720979917 33596697 PMC8573706

[B222] Sasaki K. Iwata N. Ferdousi F. Isoda H. (2019). Antidepressant‐like effect of ferulic acid via promotion of energy metabolism activity. Mol. Nutr. Food Res. 63, 1900327. 10.1002/mnfr.201900327 PMC6790570 31394019

[B223] Schimidt H. L. Garcia A. Martins A. Mello-Carpes P. B. Carpes F. P. (2017). Green tea supplementation produces better neuroprotective effects than red and black tea in Alzheimer-like rat model. Food Res. Int. 100, 442–448. 10.1016/j.foodres.2017.07.026 28873707

[B224] Schmid C. L. Streicher J. M. Meltzer H. Y. Bohn L. M. (2014). Clozapine acts as an agonist at serotonin 2A receptors to counter MK-801-induced behaviors through a βarrestin2-independent activation of Akt. Neuropsychopharmacology 39, 1902–1913. 10.1038/npp.2014.38 24531562 PMC4059899

[B225] Schopler E. Reichler R. J. Renner B. R. (2010). The childhood autism rating scale (CARS). Los Angeles, CA, USA: WPS.

[B226] Seeger T. F. Seymour P. Schmidt A. Zorn S. Schulz D. Lebel L. (1995). Ziprasidone (CP-88, 059): A new antipsychotic with combined dopamine and serotonin receptor antagonist activity. J. Pharmacol. Exp. Ther. 275, 101–113. 7562537

[B227] Shakeel S. Rehman M. U. Tabassum N. Amin U. Mir M. U. R. (2017). Effect of naringenin (a naturally occurring flavanone) against pilocarpine-induced status epilepticus and oxidative stress in mice. Pharmacogn. Mag. 13, S154–S160. 10.4103/0973-1296.203977 28479741 PMC5407108

[B228] Shao C. Yuan J. Liu Y. Qin Y. Wang X. Gu J. (2020). Epileptic brain fluorescent imaging reveals apigenin can relieve the myeloperoxidase-mediated oxidative stress and inhibit ferroptosis. Proc. Natl. Acad. Sci. U. S. A. 117, 10155–10164. 10.1073/pnas.1917946117 32327603 PMC7229752

[B229] Shapiro D. A. Renock S. Arrington E. Chiodo L. A. Liu L.-X. Sibley D. R. (2003). Aripiprazole, a novel atypical antipsychotic drug with a unique and robust pharmacology. Neuropsychopharmacology 28, 1400–1411. 10.1038/sj.npp.1300203 12784105

[B230] Sharifi-rad J. Quispe C. Herrera-Bravo J. Akram M. Abbaass W. Semwal P. (2021a). Phytochemical constituents, biological activities, and health-promoting effects of the melissa officinalis. Oxidative Med. Cell. Longev. 2021, 1–20. 10.1155/2021/6584693 PMC11283336 39071243

[B231] Sharifi-rad J. Quispe C. Herrera-Bravo J. Martorell M. Sharopov F. Tumer T. B. (2021b). A pharmacological perspective on plant-derived bioactive molecules for epilepsy. Neurochem. Res. 46, 2205–2225. 10.1007/s11064-021-03376-0 34120291

[B232] Sharifi-rad J. Quispe C. Herrera-Bravo J. Martorell M. Sharopov F. Tumer T. B. (2021c). Pharmacological perspective on plant-derived bioactive molecules for epilepsy. Neurochem. Res. 46 (9), 2205–2225. 10.1007/s11064-021-03376-0 34120291

[B233] Sharifi-rad J. Quispe C. Kumar M. Akram M. Amin M. Iqbal M. (2022). Hyssopus essential oil: An update of its phytochemistry, biological activities, and safety profile. Oxid. Med. Cell. Longev. 2022, 8442734. 10.1155/2022/8442734 35069979 PMC8776447

[B234] Sharifi-rad J. Quispe C. Patra J. K. Singh Y. D. Panda M. K. Das G. (2021d). Paclitaxel: Application in modern oncology and nanomedicine-based cancer therapy. Oxid. Med. Cell. Longev. 2021, 3687700. 10.1155/2021/3687700 34707776 PMC8545549

[B235] Shyn S. I. Hamilton S. P. (2010). The genetics of major depression: Moving beyond the monoamine hypothesis. Psychiatr. Clin. North Am. 33, 125–140. 10.1016/j.psc.2009.10.004 20159343 PMC2824618

[B236] Silva M. I. G. Silva M. A. G. DE Aquino Neto M. R. Moura B. A. DE Sousa H. L. DE Lavor E. P. H. (2009). Effects of isopulegol on pentylenetetrazol-induced convulsions in mice: Possible involvement of GABAergic system and antioxidant activity. Fitoterapia 80, 506–513. 10.1016/j.fitote.2009.06.011 19559770

[B237] Silver J. Hales R. Yudolsky S. (1990). Psychiatric consultation to neurology. Rev. Psychiatry 9.

[B238] Silver J. M. Yudofsky S. C. Hales R. E. (1991). Depression in traumatic brain injury. Neuropsychiatry, Neuropsychology, Behav. Neurology.

[B239] Silver J. M. Yudofsky S. C. Hales R. E. (1994). Neuropsychiatry of traumatic brain injury. American Psychiatric Association.

[B240] Singh D. Goel R. K. (2016). Anticonvulsant mechanism of saponins fraction from adventitious roots of Ficus religiosa: Possible modulation of GABAergic, calcium and sodium channel functions. Rev. Bras. Farmacogn. 26, 579–585. 10.1016/j.bjp.2015.10.007

[B241] Singh I. (2008). Beyond polemics: Science and ethics of ADHD. Nat. Rev. Neurosci. 9, 957–964. 10.1038/nrn2514 19020513

[B242] Smith M. T. Crouch N. R. Gericke N. Hirst M. (1996). Psychoactive constituents of the genus Sceletium NE Br. And other mesembryanthemaceae: A review. J. Ethnopharmacol. 50, 119–130. 10.1016/0378-8741(95)01342-3 8691846

[B243] Snyder S. H. Yamamura H. I. (1977). Antidepressants and the muscarinic acetylcholine receptor. Arch. Gen. Psychiatry 34, 236–239. 10.1001/archpsyc.1977.01770140126014 14603

[B244] Soofiyani S. R. Hosseini K. Forouhandeh H. Ghasemnejad T. Tarhriz V. Asgharian P. (2021). Quercetin as a novel therapeutic approach for lymphoma. Oxidative Med. Cell. Longev. 2021. 10.1155/2021/3157867 PMC8352693 34381559

[B245] Souza L. C. DE Gomes M. G. Goes A. T. Del Fabbro L. Carlos Filho B. Boeira S. P. (2013). Evidence for the involvement of the serotonergic 5-HT1A receptors in the antidepressant-like effect caused by hesperidin in mice. Prog. Neuropsychopharmacol. Biol. Psychiatry 40, 103–109. 10.1016/j.pnpbp.2012.09.003 22996046

[B317] Spina E. De Domenico P. Ruello C. Longobardo N. Gitto C. Ancione M. Di Rosa A. E. Caputi A. P. (1994). Adjunctive fluoxetine in the treatment of negative symptoms in chronic schizophrenic patients. Int. Clin. Psychopharmacol. 9 (4), 281–286. 10.1097/00004850-199400940-00007 7868850

[B246] Spinella M. (2001). The psychopharmacology of herbal medicine: Plant drugs that alter mind, brain, and behavior. MIT Press.

[B247] Stahl S. M. Grady M. M. Moret C. Briley M. (2005). SNRIs: Their pharmacology, clinical efficacy, and tolerability in comparison with other classes of antidepressants. CNS Spectr. 10, 732–747. 10.1017/s1092852900019726 16142213

[B248] Stahl S. M. Meyer J. M. (2020). The clozapine handbook. Cambridge University Press.

[B249] Stansfield R. L. (2019). When attention deficit meets the “Attention Economy”. Dissertation thesis. Available at: https://unbscholar.lib.unb.ca/islandora/object/unbscholar%3A9820

[B250] Steenkamp P. Harding N. VAN Heerden F. VAN Wyk B.-E. (2004). Fatal Datura poisoning: Identification of atropine and scopolamine by high performance liquid chromatography/photodiode array/mass spectrometry. Forensic Sci. Int. 145, 31–39. 10.1016/j.forsciint.2004.03.011 15374592

[B251] Storebø O. J. Ramstad E. Krogh H. B. Nilausen T. D. Skoog M. Holmskov M. (2015). Methylphenidate for children and adolescents with attention deficit hyperactivity disorder (ADHD). Cochrane Database Syst. Rev. 2016. 10.1002/14651858.cd009885.pub2 PMC8763351 26599576

[B252] Sundberg M. Sahin M. (2015). Cerebellar development and autism spectrum disorder in tuberous sclerosis complex. J. Child. Neurol. 30, 1954–1962. 10.1177/0883073815600870 26303409 PMC4644486

[B253] Taheri Y. Quispe C. Herrera-Bravo J. Sharifi-Rad J. Ezzat S. M. Merghany R. M. (2022). *Urtica dioica*-derived phytochemicals for pharmacological and therapeutic applications. Evid. Based. Complement. Altern. Med. 2022, 4024331. 10.1155/2022/4024331 PMC8894011 35251206

[B254] Taïwe G. S. Kuete V. (2014). Neurotoxicity and neuroprotective effects of African medicinal plants. Toxicol. Surv. Afr. Med. plants, 423–444. 10.1016/b978-0-12-800018-2.00014-5

[B255] Takeda A. Sakamoto K. Tamano H. Fukura K. Inui N. Suh S. W. (2011). Facilitated neurogenesis in the developing hippocampus after intake of theanine, an amino acid in tea leaves, and object recognition memory. Cell. Mol. Neurobiol. 31, 1079–1088. 10.1007/s10571-011-9707-0 21604187 PMC11498479

[B256] Taviano M. Miceli N. Monforte M. Tzakou O. Galati E. (2007). Ursolic acid plays a role in Nepeta sibthorpii Bentham CNS depressing effects. Phytother. Res. 21, 382–385. 10.1002/ptr.2076 17236171

[B257] Taylor G. Mcneill A. Girling A. Farley A. Lindson-Hawley N. Aveyard P. (2014). Change in mental health after smoking cessation: Systematic review and meta-analysis. Bmj 348, g1151. 10.1136/bmj.g1151 24524926 PMC3923980

[B258] Theoharides T. Asadi S. Panagiotidou S. (2012). A case series of a luteolin formulation (NeuroProtek®) in children with autism spectrum disorders. London, England: SAGE Publications Sage UK. 10.1177/039463201202500201 22697063

[B259] Tiihonen J. Mittendorfer-Rutz E. Majak M. Mehtälä J. Hoti F. Jedenius E. (2017). Real-world effectiveness of antipsychotic treatments in a nationwide cohort of 29 823 patients with schizophrenia. JAMA psychiatry 74, 686–693. 10.1001/jamapsychiatry.2017.1322 28593216 PMC5710250

[B260] Trebatická J. Kopasová S. Hradečná Z. Činovský K. Škodáček I. Šuba J. . 2006. Treatment of ADHD with French maritime pine bark extract, Pycnogenol®. Eur. Child. Adolesc. Psychiatry, 15, 329–335,. 10.1007/s00787-006-0538-3 16699814

[B261] Trofor L. Crisan-Dabija R. Cioroiu M. E. Man M. A. Cioroiu I. B. Buculei I. (2020). Evaluation of oxidative stress in smoking and NON-smoking patients diagnosed with anxious-depressive disorder. Farmacia 68, 82–89. 10.31925/farmacia.2020.1.12

[B262] Tsilioni I. Taliou A. Francis K. Theoharides T. (2015). Children with autism spectrum disorders, who improved with a luteolin-containing dietary formulation, show reduced serum levels of TNF and IL-6. Transl. Psychiatry 5, e647. 10.1038/tp.2015.142 26418275 PMC5545641

[B263] Tsoukalas D. Buga A. M. Docea A. O. Sarandi E. Mitrut R. Renieri E. (2021). Reversal of brain aging by targeting telomerase: A nutraceutical approach. Int. J. Mol. Med. 48, 199. 10.3892/ijmm.2021.5032 34515324 PMC8448543

[B264] Tuchman R. Cuccaro M. Alessandri M. (2010). Autism and epilepsy: Historical perspective. Brain Dev. 32, 709–718. 10.1016/j.braindev.2010.04.008 20510557

[B265] Uebel-von sandersleben H. Rothenberger A. Albrecht B. Rothenberger L. G. Klement S. Bock N. (2014). “Ginkgo biloba extract EGb 761® in children with ADHD,” in Zeitschrift für Kinder-und Jugendpsychiatrie und Psychotherapie. 10.1024/1422-4917/a000309 25163996

[B266] Uk N. C. G. C. (2012). The epilepsies: The diagnosis and management of the epilepsies in adults and children in primary and secondary care.

[B267] Underwood B. R. Imarisio S. Fleming A. Rose C. Krishna G. Heard P. (2010). Antioxidants can inhibit basal autophagy and enhance neurodegeneration in models of polyglutamine disease. Hum. Mol. Genet. 19, 3413–3429. 10.1093/hmg/ddq253 20566712 PMC2916709

[B268] Urdaneta K. E. Castillo M. A. Montiel N. Semprún-Hernández N. Antonucci N. Siniscalco D. (2018). Autism spectrum disorders: Potential neuro-psychopharmacotherapeutic plant-based drugs. Assay. Drug Dev. Technol. 16, 433–444. 10.1089/adt.2018.848 30427697

[B269] Van os J. Kapur S. (2009). Schizophrenia. Lancet 374, 635–645. 10.1016/S0140-6736(09)60995-8 19700006

[B270] Verrotti A. Tocco A. Salladini C. Latini G. Chiarelli F. (2005). Human photosensitivity: From pathophysiology to treatment. Eur. J. Neurol. 12, 828–841. 10.1111/j.1468-1331.2005.01085.x 16241971

[B271] Vlad R. Golu F. Toma A. Draganescu D. Oprea B. Chiper B. I. (2020). Depression and anxiety in Romanian medical students: Prevalence and associations with personality. Farmacia 68, 944–949. 10.31925/farmacia.2020.5.24

[B272] Walsh C. A. Morrow E. M. Rubenstein J. L. (2008). Autism and brain development. Cell 135, 396–400. 10.1016/j.cell.2008.10.015 18984148 PMC2701104

[B273] Wang J. Ferruzzi M. G. Ho L. Blount J. Janle E. M. Gong B. (2012). Brain-targeted proanthocyanidin metabolites for Alzheimer's disease treatment. J. Neurosci. 32, 5144–5150. 10.1523/JNEUROSCI.6437-11.2012 22496560 PMC3348654

[B274] Wang R. Li Y.-B. Li Y.-H. Xu Y. Wu H.-L. Li X.-J. (2008). Curcumin protects against glutamate excitotoxicity in rat cerebral cortical neurons by increasing brain-derived neurotrophic factor level and activating TrkB. Brain Res. 1210, 84–91. 10.1016/j.brainres.2008.01.104 18420184

[B275] Wang R. Li Y.-H. Xu Y. Li Y.-B. Wu H.-L. Guo H. (2010). Curcumin produces neuroprotective effects via activating brain-derived neurotrophic factor/TrkB-dependent MAPK and PI-3K cascades in rodent cortical neurons. Prog. Neuropsychopharmacol. Biol. Psychiatry 34, 147–153. 10.1016/j.pnpbp.2009.10.016 19879308

[B276] Wang R. Yan H. Tang X. C. (2006). Progress in studies of huperzine A, a natural cholinesterase inhibitor from Chinese herbal medicine. Acta Pharmacol. Sin. 27, 1–26. 10.1111/j.1745-7254.2006.00255.x 16364207

[B277] Wasilewska J. Klukowski M. (2015). Gastrointestinal symptoms and autism spectrum disorder: Links and risks–a possible new overlap syndrome. Pediatr. Health Med. Ther. 6, 153–166. 10.2147/PHMT.S85717 PMC5683266 29388597

[B278] Wattanathorn J. Chonpathompikunlert P. Muchimapura S. Priprem A. Tankamnerdthai O. (2008). Piperine, the potential functional food for mood and cognitive disorders. Food Chem. Toxicol. 46, 3106–3110. 10.1016/j.fct.2008.06.014 18639606

[B279] Weissman M. M. Olfson M. (1995). Depression in women: Implications for health care research. Science 269, 799–801. 10.1126/science.7638596 7638596

[B280] Wilens T. E. Spencer T. J. (2010). Understanding attention-deficit/hyperactivity disorder from childhood to adulthood. Postgrad. Med. 122, 97–109. 10.3810/pgm.2010.09.2206 20861593 PMC3724232

[B281] Willcutt E. G. (2012). The prevalence of DSM-IV attention-deficit/hyperactivity disorder: A meta-analytic review. Neurotherapeutics 9, 490–499. 10.1007/s13311-012-0135-8 22976615 PMC3441936

[B282] Willner P. Scheel-Krüger J. Belzung C. (2013). The neurobiology of depression and antidepressant action. Neurosci. Biobehav. Rev. 37, 2331–2371. 10.1016/j.neubiorev.2012.12.007 23261405

[B283] Woo T. S. Yoon S. Y. Cheong J. H. Choi J. Y. Lee H. L. Choi Y. J. (2011). Anticonvulsant effect of Artemisia capillaris Herba in mice. Biomol. Ther. Seoul. 19, 342–347. 10.4062/biomolther.2011.19.3.342

[B284] Wood J. J. Drahota A. Sze K. Har K. Chiu A. Langer D. A. (2009). Cognitive behavioral therapy for anxiety in children with autism spectrum disorders: A randomized, controlled trial. J. Child. Psychol. Psychiatry 50, 224–234. 10.1111/j.1469-7610.2008.01948.x 19309326 PMC4231198

[B285] Woodward K. (2015). Psychosocial studies: An introduction. Routledge.

[B286] Wu E. Q. Shi L. Birnbaum H. Hudson T. Kessler R. (2006). Annual prevalence of diagnosed schizophrenia in the USA: A claims data analysis approach. Psychol. Med. 36, 1535–1540. 10.1017/S0033291706008191 16907994

[B287] Wu J. Chen H. Li H. Tang Y. Yang L. Cao S. (2016). Antidepressant potential of chlorogenic acid-enriched extract from Eucommia ulmoides Oliver bark with neuron protection and promotion of serotonin release through enhancing synapsin I expression. Molecules 21, 260. 10.3390/molecules21030260 26927040 PMC6274286

[B288] Wulff K. Donato D. Lurie N. (2015). What is health resilience and how can we build it? Annu. Rev. Public Health 36, 361–374. 10.1146/annurev-publhealth-031914-122829 25581148

[B321] Wynn J. K. Green M. F. Hellemann G. Karunaratne K. Davis M. C. Marder S. R. (2018). The effects of curcumin on brain-derived neurotrophic factor and cognition in schizophrenia: A randomized controlled study. Schizophr. Res. 195, 572–573. 10.1016/j.schres.2017.09.046 28965778

[B289] Xu N. Li X. Zhong Y. (2015). Inflammatory cytokines: potential biomarkers of immunologic dysfunction in autism spectrum disorders. Mediators Inflamm. 2015, 531518. 10.1155/2015/531518 25729218 PMC4333561

[B290] Xu Y. Ku B.-S. Yao H.-Y. Lin Y.-H. Ma X. Zhang Y.-H. (2005a). Antidepressant effects of curcumin in the forced swim test and olfactory bulbectomy models of depression in rats. Pharmacol. Biochem. Behav. 82, 200–206. 10.1016/j.pbb.2005.08.009 16171853

[B291] Xu Y. Ku B.-S. Yao H.-Y. Lin Y.-H. Ma X. Zhang Y.-H. (2005b). The effects of curcumin on depressive-like behaviors in mice. Eur. J. Pharmacol. 518, 40–46. 10.1016/j.ejphar.2005.06.002 15987635

[B292] Xu Y. Ku B. Cui L. Li X. Barish P. A. Foster T. C. (2007). Curcumin reverses impaired hippocampal neurogenesis and increases serotonin receptor 1A mRNA and brain-derived neurotrophic factor expression in chronically stressed rats. Brain Res. 1162, 9–18. 10.1016/j.brainres.2007.05.071 17617388

[B293] Xu Y. Li S. Chen R. Li G. Barish P. A. You W. (2010a). Antidepressant-like effect of low molecular proanthocyanidin in mice: Involvement of monoaminergic system. Pharmacol. Biochem. Behav. 94, 447–453. 10.1016/j.pbb.2009.10.007 19857512

[B294] Xu Y. Wang Z. You W. Zhang X. Li S. Barish P. A. (2010b). Antidepressant-like effect of trans-resveratrol: Involvement of serotonin and noradrenaline system. Eur. Neuropsychopharmacol. 20, 405–413. 10.1016/j.euroneuro.2010.02.013 20353885

[B295] Yáñez M. Fraiz N. Cano E. Orallo F. (2006). Inhibitory effects of cis-and trans-resveratrol on noradrenaline and 5-hydroxytryptamine uptake and on monoamine oxidase activity. Biochem. Biophys. Res. Commun. 344, 688–695. 10.1016/j.bbrc.2006.03.190 16631124

[B296] Yao X. Li L. Kandhare A. D. Mukherjee-Kandhare A. A. Bodhankar S. L. (2020). Attenuation of reserpine-induced fibromyalgia via ROS and serotonergic pathway modulation by fisetin, a plant flavonoid polyphenol. Exp. Ther. Med. 19, 1343–1355. 10.3892/etm.2019.8328 32010308 PMC6966137

[B297] Yeni Y. Cakir Z. Hacimuftuoglu A. Taghizadehghalehjoughi A. Okkay U. Genc S. (2022). A selective histamine H4 receptor antagonist, JNJ7777120, role on glutamate transporter activity in chronic depression. J. Pers. Med. 12, 246. 10.3390/jpm12020246 35207733 PMC8880293

[B298] Yi L.-T. Xu H.-L. Feng J. Zhan X. Zhou L.-P. Cui C.-C. (2011). Involvement of monoaminergic systems in the antidepressant-like effect of nobiletin. Physiol. Behav. 102, 1–6. 10.1016/j.physbeh.2010.10.008 20951716

[B299] Yoon S. Y. DELA Peña I. C. Shin C. Y. Son K. H. Lee Y. S. Ryu J. H. (2011). Convulsion-related activities of Scutellaria flavones are related to the 5, 7-dihydroxyl structures. Eur. J. Pharmacol. 659, 155–160. 10.1016/j.ejphar.2011.03.012 21440538

[B300] Yoshino S. Hara A. Sakakibara H. Kawabata K. Tokumura A. Ishisaka A. (2011). Effect of quercetin and glucuronide metabolites on the monoamine oxidase-A reaction in mouse brain mitochondria. Nutrition 27, 847–852. 10.1016/j.nut.2010.09.002 21371861

[B301] Yu Y.-H. Xie W. Bao Y. Li H.-M. Hu S.-J. Xing J.-L. (2012). Saikosaponin a mediates the anticonvulsant properties in the HNC models of AE and SE by inhibiting NMDA receptor current and persistent sodium current. PLoS One 7, e50694. 10.1371/journal.pone.0050694 23209812 PMC3510157

[B302] Yudofsky S. C. Hales R. E. (2002). Neuropsychiatry and the future of psychiatry and neurology. Am. J. Psychiatry 159, 1261–1264. 10.1176/appi.ajp.159.8.1261 12153815

[B314] Yusha'u Y. Muhammad U. A. Nze M. Egwuma J. M. Igomu O. J. Abdulkadir M. (2017). Modulatory Role of Rutin Supplement on Open Space Forced Swim Test Murine Model of Depression. Niger. J. Physiol. Sci. 32 (2), 201–205. 29485642

[B303] Zangara A. (2003). The psychopharmacology of huperzine A: An alkaloid with cognitive enhancing and neuroprotective properties of interest in the treatment of alzheimer's disease. Pharmacol. Biochem. Behav. 75, 675–686. 10.1016/s0091-3057(03)00111-4 12895686

[B304] Zeni A. L. B. Zomkowski A. D. E. Maraschin M. Rodrigues A. L. S. Tasca C. I. (2012). Ferulic acid exerts antidepressant-like effect in the tail suspension test in mice: Evidence for the involvement of the serotonergic system. Eur. J. Pharmacol. 679, 68–74. 10.1016/j.ejphar.2011.12.041 22266492

[B305] Zhang F. Lu Y.-F. Wu Q. Liu J. Shi J.-S. (2012). Resveratrol promotes neurotrophic factor release from astroglia. Exp. Biol. Med. 237, 943–948. 10.1258/ebm.2012.012044 22875340

[B306] Zhang L. Xu T. Wang S. Yu L. Liu D. Zhan R. (2013). NMDA GluN2B receptors involved in the antidepressant effects of curcumin in the forced swim test. Prog. Neuropsychopharmacol. Biol. Psychiatry 40, 12–17. 10.1016/j.pnpbp.2012.08.017 22960607

[B307] Zhang Z.-J. (2004). Therapeutic effects of herbal extracts and constituents in animal models of psychiatric disorders. Life Sci. 75, 1659–1699. 10.1016/j.lfs.2004.04.014 15268969

[B308] Zhen L. Zhu J. Zhao X. Huang W. An Y. Li S. (2012). The antidepressant-like effect of fisetin involves the serotonergic and noradrenergic system. Behav. Brain Res. 228, 359–366. 10.1016/j.bbr.2011.12.017 22197297

[B309] Zheng L. T. Ock J. Kwon B.-M. Suk K. (2008). Suppressive effects of flavonoid fisetin on lipopolysaccharide-induced microglial activation and neurotoxicity. Int. Immunopharmacol. 8, 484–494. 10.1016/j.intimp.2007.12.012 18279803

[B310] Zhu H. L. Wan J. B. Wang Y. T. Li B. C. Xiang C. He J. (2014). Medicinal compounds with antiepileptic/anticonvulsant activities. Epilepsia 55, 3–16. 10.1111/epi.12463 24299155

[B311] Zuiki M. Chiyonobu T. Yoshida M. Maeda H. Yamashita S. Kidowaki S. (2017). Luteolin attenuates interleukin-6-mediated astrogliosis in human iPSC-derived neural aggregates: A candidate preventive substance for maternal immune activation-induced abnormalities. Neurosci. Lett. 653, 296–301. 10.1016/j.neulet.2017.06.004 28595950

